# B‐Site‐Metal‐Mediated Coke‐Resistant CO_2_ Electrolysis on Perovskite Surfaces

**DOI:** 10.1002/advs.202503970

**Published:** 2025-05-21

**Authors:** Tongbao Wang, Yu Mao, Pengfei Ou, Zhijie Wang, Yifan Li, Hao Li, Binbin Pan, Ximeng Lv, Yanguang Li, Gengfeng Zheng, Chengzhi Guan, Yi Cui, Ziyun Wang, Yuhang Wang

**Affiliations:** ^1^ State Key Laboratory of Bioinspired Interfacial Materials Science Institute of Functional Nano & Soft Materials (FUNSOM) Soochow University 199 Ren'ai Road Suzhou Jiangsu 215123 China; ^2^ Jiangsu Key Laboratory for Advanced Negative Carbon Technologies Soochow University 199 Ren'aif Road Suzhou Jiangsu 215123 China; ^3^ School of Chemical Sciences The University of Auckland 23 Symonds Street Auckland 1010 New Zealand; ^4^ Department of Chemistry National University of Singapore 3 Science Drive 3 Singapore 117543 Singapore; ^5^ Shanghai Institute of Applied Physics Chinese Academy of Sciences 2019 Jialuo Road Shanghai 201800 China; ^6^ i‐LAB Vacuum Interconnected Nanotech Workstation Suzhou Institute of Nano‐Tech and Nano‐Bionics Chinese Academy of Sciences 398 Ruoshui Road Suzhou 215123 China; ^7^ Laboratory of Advanced Materials Department of Chemistry Fudan University 2005 Songhu Road Shanghai 200438 China

**Keywords:** coke resistance, electrochemical CO_2_ reduction, high CO_2_ conversion, perovskite oxides, solid oxide electrolysis

## Abstract

High‐temperature CO_2_ reduction to CO using perovskite‐oxide‐based solid oxide electrochemical cells holds promise for carbon‐neutral chemical production, yet currently faces the challenge of coke formation that leads to device failure. A key reason behind this challenge is the absence of a correlation between the coke formation mechanism and perovskite structures. Here, lanthanum strontium cobalt ferrite perovskites are taken with a classical ABO_3_ structure as examples to study coke formation on them and unravel the dependence of coke resistance on the Fe stoichiometry. Lowering the Co versus Fe ratio suppresses B‐site metal exsolution, and thus, coke formation is catalyzed by these metals/alloys. Using (La_0.6_Sr_0.4_)_0.95_Co_0.2_Fe_0.8_O_3‐δ_ as an example, this study reports an outlet CO pressure of 0.86 ± 0.02 atm at 800 °C, closely approaching the thermodynamic threshold for coking. The cell offers a stable outlet CO pressure of ≈0.8 atm in 320‐h electrolysis at 220 mA cm^−2^ and the potential to build a high‐performance tandem system for efficient electrosynthesis of multi‐carbon products from CO_2_.

## Graphical abstract

This study reveals the correlation between the B‐site metals of La_0.6_Sr_0.4_Co_1‐x_Fe_x_O_3‐δ_ (LSC_1‐x_F_x_) and their coke resistance during high‐temperature CO_2_ electrolysis. Elevated Fe/Co ratios enhance the coke resistance by suppressing excessive oxygen vacancy formation and Co/Fe exsolution. This underlying mechanism enables highly stable CO_2_ electrolysis performance and a tandem reactor for multi‐carbon product synthesis with high CO_2_ utilization efficiency.

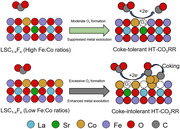

## 1 Introduction

Renewable‐energy‐powered carbon dioxide (CO_2_) electroreduction offers a route to decarbonize chemical manufacturing. High‐temperature CO_2_ reduction (HT‐CO_2_RR) to CO using solid oxide electrochemical cells (SOECs) is of great interest in light of its high activity and high energy efficiency.^[^ ^1^, ^2^, ^3^ ^]^ This technology, coupled with CO upgrading technologies, e.g., CO hydrogenation or electroreduction, produces carbon‐neutral and value‐added hydrocarbons and oxygenates.^[^ ^4^ ^]^

Nevertheless, in today's HT‐CO_2_RR, unwanted coke formation originating from CO disproportionation (2CO ↔ C + CO_2_) causes a decline in performance after a typical 3 h of operation at a 500 mA cm^−2^ current density.^[^ ^5^ ^]^ Coke formation imposes thermodynamic limits on CO concentration during HT‐CO_2_RR at different temperatures, causing a high risk of cell failure during potential operation accidents. Using coking‐active catalysts (e.g., Ni) further reduces these limits,^[^ ^6^, ^7^, ^8^ ^]^ even when the electrode porosity,^[^ ^9^ ^]^ morphology,^[^ ^10^ ^]^ and composition are optimized.^[^ ^11^, ^12^ ^]^ The practical solution to the coke formation issue is to reduce the CO partial pressure to 0.45 atm,^5^ which inevitably increases the downstream CO separation cost.^[^ ^13^, ^14^, ^15^ ^]^

Recently, catalysts featuring surface oxygen vacancies (O_v_) were associated with coke tolerance in HT‐CO_2_RR.^[^ ^5^ ^]^ Perovskite oxides, exemplified by La_0.4_Sr_0.6_Co_0.2_Fe_0.7_Mo_0.1_O_3‐δ_, Sr_2_Fe_1.4_Ru_0.1_Mo_0.5_O_6−δ_, Sr_2_Fe_1.45_Ir_0.05_Mo_0.5_O_6‐δ_, etc., are a category of materials with surface O_v_ and exhibit high CO_2_ electrolysis activities.^[^ ^16^, ^17^, ^18^, ^19^ ^]^ These materials often undergo reconstruction driven by B‐site metal exsolution and the formation of interfaces between coking‐active metals and perovskites during CO_2_ electrolysis.^[^ ^20^, ^21^, ^22^, ^23^, ^24^ ^]^ Unfortunately, determinations of coke formation dynamics on perovskite‐based cathodes and its correlation with perovskite structure evolution under HT‐CO_2_RR operating conditions are absent. Consequently, the outlet CO pressures (P_CO_), the indicator of coke resistance, are far below the thermodynamic limit (≤0.2 atm in previous reports vs ≥ 0.78 atm in theory) caused by coke formation at typical operating temperatures of 750–850 °C in present‐day SOECs based on perovskite cathodes.^[^ ^5^, ^6^, ^7^, ^8^, ^20^, ^21^, ^22^, ^23^ ^]^

Here, taking lanthanum strontium cobalt ferrite perovskites with various B‐site Co versus Fe ratios (i.e., LSC_1‐x_F_x_, x = 0, 0.2, 0.5, 0.6, 0.7, 0.8, and 1) as examples, we find, using electrochemical measurements, in situ near ambient pressure X‐ray photoelectron spectroscopy (NAP‐XPS) and quasi‐in‐situ Raman spectroscopy, a positive correlation between coke resistance and Fe:Co ratio at the B site of the perovskite, and a lack of dependence on surface O_v_ concentration and metal‐oxide interface densities. We obtained anti‐coking HT‐CO_2_RR with an outlet CO pressure (P_CO_) limit of ≈0.85 atmospheres (atm) using LSC_0.2_F_0.8_ and LSF perovskite oxide catalysts, closely approaching the thermodynamic threshold for coke formation at 800 °C. The coke resistance increases by a factor of ≈1.4 compared to commercial Ni/yttria‐stabilized ZrO_2_ (comm‐Ni/YSZ) SOEC controls. Computational studies suggest a role for the Fe:Co ratio at the B‐site of the perovskite oxide: high Fe:Co ratios inhibit B‐site‐metal exsolution that would otherwise form coking‐active metals and alloys. As a result, we achieve a CO Faradaic efficiency of ≈100% and an electrolysis energy efficiency (EE) of ≈74%: this is sustained over 320‐h operation at 220 mA cm^−2^ and 0.8 atm P_CO_ with a cell degradation rate of less than 0.09 mV h^−1^. We devise a tandem system enabling a CO_2_ SPU of 57% ± 2% to multi‐carbon (C_2+_) products.

## 2 Results and Discussion

### 2.1 HT‐CO_2_RR Measurements

**Figure 1:**
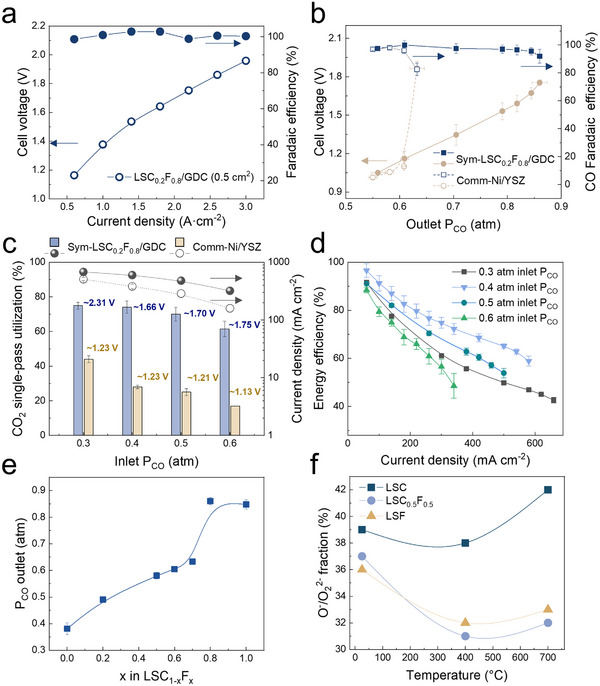
The performance metrics for high‐temperature CO_2_‐to‐CO electrolysis at 800 °C. a) The cell voltage of CO FE in the current density range of 0.5–3 A cm^−2^ on 0.5‐cm^2^ sym‐LSCF/GDC button cells. b) Comparison of the cell voltages (left y‐axis) and the CO FEs (right y‐axis) at different outlet P_CO_ on 5‐cm^2^ sym‐LSC_0.2_F_0.8_/GDC and 3.14‐cm^2^ comm‐Ni/YSZ cells. c) The CO_2_ SPU (left y‐axis) and current density (right y‐axis) at different inlet P_CO_ on 5‐cm^2^ sym‐LSC_0.2_F_0.8_/GDC and 3.14‐cm^2^ comm‐Ni/YSZ cells. Labels denote operating cell voltages correlated with conversion rates, with yellow representing Ni‐YSZ and blue indicating sym‐LSC_0.2_F_0.8_/GDC. d) The CO electrolysis EEs obtained at 5‐cm^2^ sym‐LSC_0.2_F_0.8_/GDC cells operated at current densities from 60 to 660 mA cm^−2^ and under various inlet P_CO_. Error bars correspond to standard deviations of three independent measurements. e) Comparison of coke resistance (i.e., the outlet P_CO_) of different 5‐cm^2^ LSC_1‐x_F_x_. The total flow rates of CO_2_ and CO for these measurements are 50 sccm. f) Comparison of surface O_v_ fractions on LSC_1‐x_F_x_ at various temperatures.

We fabricated LSC_0.2_F_0.8_‐based SOECs having a cell‐symmetric architecture in which both the cathode and anode are LSC_0.2_F_0.8_ catalysts mixed with gadolinium‐doped CeO_2_ (GDC), a high‐performance oxygen‐conductive material.^[^ ^25^, ^26^ ^]^ 200‐µm‐thick scandia‐stabilized ZrO_2_ (SSZ) disks were used as the electrolyte supports (Figure S1a, Supporting Information). From SEM, the LSCF‐GDC cathode porosity is ≈13% (Figure S1b, Supporting Information). To avoid polarization‐induced rapid cell degradation, we used as feedstock a CO/CO_2_ mixture with selected P_CO_ to detect the onset of coke formation on LSCF‐GDC and Ni‐YSZ (coke formation causes cell delamination and the consequent increase in the cell resistance leads to a runaway increase in cell voltage).^[^ ^5^, ^6^, ^7^, ^9^ ^]^ The symmetric LSC_0.2_F_0.8_/GDC (sym‐LSC_0.2_F_0.8_/GDC) cell with an active area of 0.5 cm^2^ offers 1.31 A cm^−2^ at 1.5 V during HT‐CO_2_RR (**Figure** **1**a; Figure S2, Supporting Information). When scaled to 5 cm^2^ and evaluated at 540 mA cm^−2^, the cell achieves an outlet P_CO_ of 0.88 atm, equivalent to the thermodynamic threshold for coke formation at 800 °C. At this outlet P_CO_, the sym‐LSC_0.2_F_0.8_/GDC cell has a stable cell voltage of ≈1.8 V for an initial 40 min (Figure S3, Supporting Information). By contrast, the outlet P_CO_ for the comm‐Ni‐YSZ cell is limited to below 0.65 atm, seen in the sharp rise of cell voltage from 1.2 to 1.8 V after 1500 s (Figure S3, Supporting Information).

We compared the coke resistance of sym‐LSC_0.2_F_0.8_/GDC and comm‐Ni/YSZ cells at different inlet P_CO_ from 0.30 to 0.60 atm (Figure 1c; Figure S4a–g, Supporting Information). The current density was increased stepwise until rapid cell voltage increase was recorded (Figure S4a–g, Supporting Information). At an inlet P_CO_ of 0.3 atm and a current density of 660 mA cm^−2^, the CO_2_ SPU reaches 75% ± 2%, >1.6× than the maximum for comm‐Ni/YSZ (Figure 1c). The electrolysis (without considering heating) and heat‐included EE are >80% and >70%, respectively, at 100 mA cm^−2^. These are achieved for a wide range of inlet P_CO_ and remain higher than 50% at 300 mA cm^−2^ (Figure 1d; Figure S4h, Supporting Information). We further screened the HT‐CO_2_RR performance on electrodes with various Fe:Co ratios (Figures S5,S6a–c,g, and S7, Supporting Information). The highest outlet P_CO_ on LSC_1‐x_F_x_/GDC positively correlates with the Fe:Co ratio. LSC_0.2_F_0.8_/GDC and LSF/GDC achieve 0.86 ± 0.02 and 0.84 ± 0.02 atm, respectively (Figure 1e; Figures S6d–f,h,i, S7a–c, and S8a–c, Supporting Information). The parent LSC_1‐x_F_x_ perovskite oxides retain their structural integrities after HT‐CO_2_RR measurements (Figures S9a–c and S10, Supporting Information), which aligns with the literature reports.^[^ ^16^ ^]^

To exclude the contribution of GDC, a known anti‐coking electrolyte material, we fabricated sym‐LSC_0.2_F_0.8_ (GDC‐free) cells using La_0.8_Sr_0.2_Ga_0.8_Mg_0.2_O_3‐δ_ (LSGM) electrolyte supports and Ni/GDC cells. Under HT‐CO_2_RR conditions, the sym‐LSC_0.2_F_0.8_/GDC cell and the sym‐LSC_0.2_F_0.8_ cell demonstrate higher coke resistance (≈0.85 atm for sym‐LSC_0.2_F_0.8_/GDC and ≈0.75 atm for sym‐LSC_0.2_F_0.8_) compared to the comm‐Ni/YSZ and Ni/GDC cell (≤0.65 atm for both) (Figure 1b; Figures S4e–g, S11, and S12, Supporting Information). The dependence of coke resistance on O_v_ concentrations of LSC_1‐x_F_x_ was also ruled out by the absence of a correlation between the experimentally measured O_v_ concentration and the maximum outlet P_CO_ (Figure 1f; Figure S13, Supporting Information). Moreover, H_2_/Ar treated LSC_0.2_F_0.8_ presents an increased O_v_ concentration but reduced outlet P_CO_ (Figure S14, Supporting Information), further corroborating the more impactful role of B‐site metals than surface O_v_ in determining coke resistance in the case of LSC_1‐x_F_x_. The distribution function of relaxation times (DRT) analysis reveals that the electrochemical adsorption/dissociation process (P4) dominates CO_2_ electrolysis kinetics for all LSC_1‐x_F_x_ samples (Figure S15, Supporting Information), confirming that the difference in coke resistance for these perovskites does not stem from the change in rate‐limiting steps. In addition, Sr segregation was seen on LSC, LSC_0.5_F_0.5_, and LSF after HT‐CO_2_RR, indicating its irrelevance to coke resistance on LSC_1‐x_F_x_ (Figure S16 and Table S1, Supporting Information).

### 2.2 Evidence of the Correlation Between the Fe:Co Ratio and Coke Resistance

We then studied the origins of coke resistance in the LSC_0.2_F_0.8_/GDC electrode by investigating the cell following HT‐CO_2_RR. Microscopy of the LSC_0.2_F_0.8_/GDC after the 40‐min operation at 540 mA cm^−2^ and a 0.88 atm outlet P_CO_ shows carbon nanotubes at electrolyte/electrode interfaces (Figure S1c,d, Supporting Information), leading to the delamination of the electrode layer (Figure S1c, Supporting Information). CoFe nanoparticle exsolution on LSC_0.2_F_0.8_ surfaces was also observed, consistent with the literature (Figure S1e–g, Supporting Information).^[^ ^16^ ^]^ For comm‐Ni/YSZ cells, similar delamination was observed at 0.65 atm P_CO_ (Figure S17, Supporting Information), consistent with carbon deposition catalyzed by oxide‐derived Ni, as seen in previous reports.^[^ ^5^, ^6^, ^11^, ^27^ ^]^ Using ex‐situ Raman spectroscopy, we found that the onset P_CO_ for coke formation is 0.85 atm on LSC_0.2_F_0.8_/GDC (Figure S1h, Supporting Information), while it decreases to 0.64 atm on Ni/YSZ and 0.40 atm on LSC/GDC (Figures S8a and S17f, Supporting Information).

**Figure 2:**
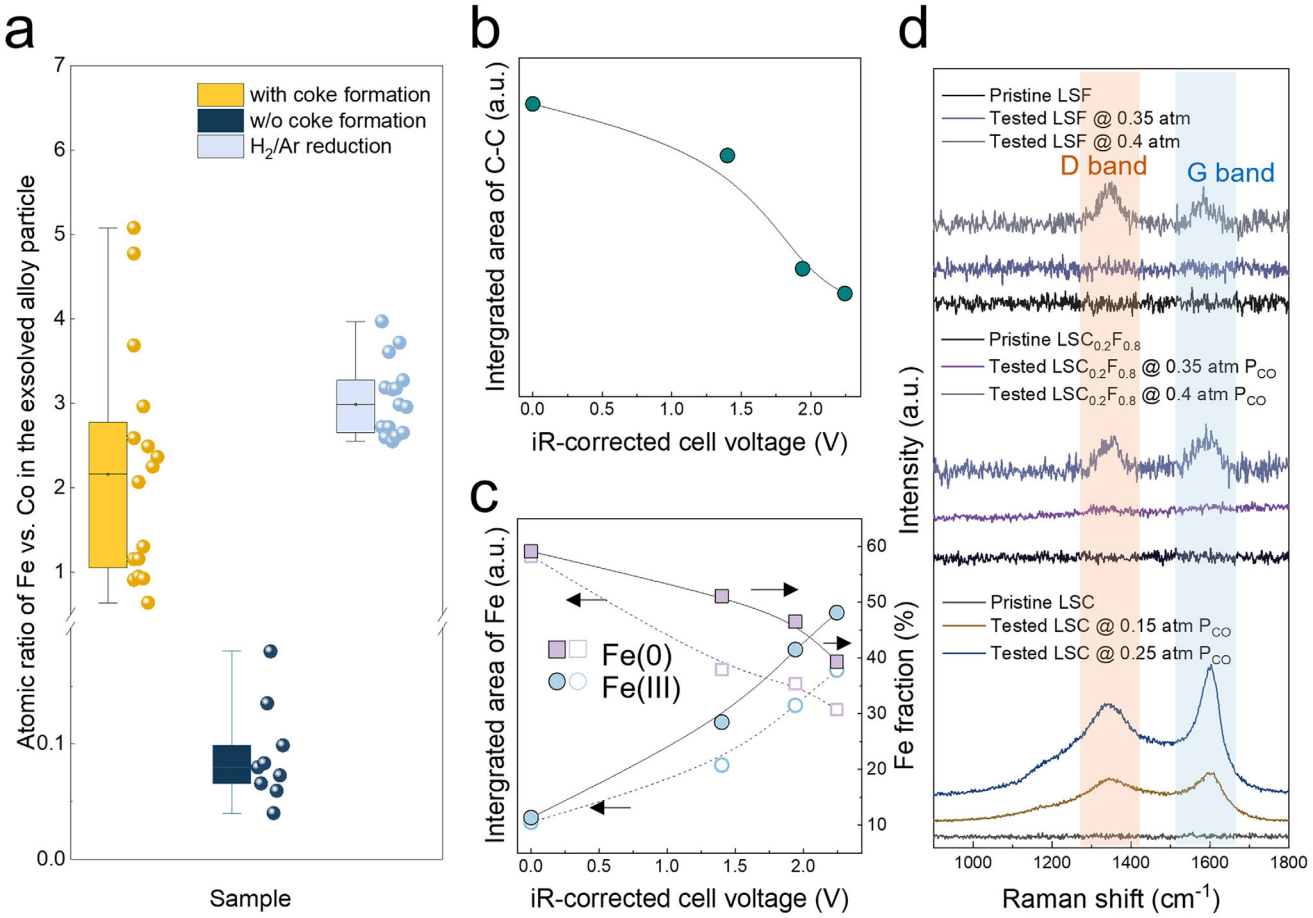
Mechanistic studies using NAP‐XPS and Raman spectroscopies. a) The Fe:Co ratios in exsolved CoFe nanoparticles under different conditions (with, without (w/o) coke formation, and reduced in 10% H_2_/Ar). b) The integrated C‐C peak area. c) The integrated peak area (left y‐axis) and the fraction (right y‐axis) of Fe(0) and Fe(III). All spectra were collected at different applied potentials, including OCV, at 0.45 mbar P_CO_ and 0.15 mbar P_CO2_. d) Comparison of coke tolerance on LSC, LSC_0.2_F_0.8_, and LSF using the quasi‐in‐situ Raman study. NAP‐XPS measurements were performed at 700 °C. The temperature for quasi‐in‐situ Raman studies was 650 °C, and the total flow rate of CO_2_ and CO was 50 sccm.

To identify the catalyst for coke formation when LSC_0.2_F_0.8_/GDC serves as the cathode, we carried out transmission electron microscopy (TEM) and energy dispersive spectroscopy (EDS) studies. We found a correlation between the Fe:Co ratio in the exsolved CoFe nanoparticles and coke formation. For the samples with coke formation, graphite carbon encapsulated CoFe nanoparticles with Fe:Co ratios of >2.8:1 were seen regardless of the applied voltages (**Figure** **2**a; Figure S18–S20, and Tables S2 and S3, Supporting Information). However, coke‐free tests were linked with much lower Fe:Co ratios of >1:11 (Figure 2a; Figure S21–S25, and Tables S2 and S4, Supporting Information). Such phenomena were constantly observed at the relatively low current densities shown in Figure S4 (Supporting Information) and high current densities greater than 1.8 A cm^−2^ (Figure S2a,b, Supporting Information). To ascertain the contribution of reducing conditions to the equilibrium Fe:Co ratio, we treated an as‐made sym‐LSC_0.2_F_0.8_/GDC cell under 10% H_2_/Ar atmosphere, a condition comparably reducing to HT‐CO_2_RR but being substantially free of O‐containing reactant, and obtained CoFe particles with a Fe:Co ratio of ≈3:1 (Figure 2a; Figure S26 and Table S5, Supporting Information), comparable to that obtained under coke formation conditions.

We studied this correlation further using in situ NAP‐XPS and quasi‐in‐situ Raman spectroscopy (Figure 2b‐d; Figures S8, S27–S30, and Table S7, Supporting Information). We first looked at C 1s, Fe 2p, and Co 2p spectra under 0.3 mbar P_CO_ and 0.3 mbar P_CO2_ (P_CO_:P_CO2_ = 1:1), under which coke does not form without applied bias at testing temperature ≈700 °C. When the applied bias increases from the open‐circuit voltage (OCV) to ≈2.7 V, the current increases stepwise to ≈100 mA (Figure S27c, Supporting Information), and the spectra shift to low‐binding‐energy regimes (Figure S28a, Supporting Information). During measurement, we saw a coke‐free process at each applied potential from the C 1s spectra, on which the C‐C sp^3^ peak centered at 284.6 eV is absent (Figure S28b, Supporting Information). The Fe 2p spectra show metallic Fe (at Fe 2p 3/2 ≈706.9 eV), confirming the formation of CoFe alloy nanoparticles (Figure S28c,d, Supporting Information). The low intensities of the Co 2p spectra caused by the low concentration of Co in LSC_0.2_F_0.8_ prevent us from accurate spectrum deconvolution. The metallic Fe fraction is ≈5% and near constant with applied voltage variation. Given that the exsolved CoFe has low Fe:Co ratios (Figures S21–S25, Tables S2, and S4, Supporting Information), the perovskite is likely to possess a higher surface concentration of Co than Fe, and only small fractions of B‐site cations are reduced to metal under coke‐free HT‐CO_2_RR conditions.

We then surveyed the C 1s, Fe 2p, and Co 2p spectra under 0.45 mbar P_CO_ and 0.15 mbar P_CO2_ (P_CO_:P_CO2_ = 3:1), a condition that causes coke formation at OCV (Figure 2b; Figure S29a,c, Supporting Information). We saw a decreased total area of the C‐C sp^3^ and C‐C sp^2^, along with increased cathodic overpotential (Figure 2b; Figure S29c, Supporting Information). Coke formation at OCV drives the exsolution of the B‐site metals, proved by the escalation of Fe(0) fraction by a factor of ≈10 (≈6% ± 1% when P_CO_:P_CO2_ = 1:1 vs ≈60% when P_CO_:P_CO2_ = 3:1). This also indicates that the chemical reaction between LSC_1‐x_F_x_ and coke is a stronger driving force for B‐site metal exsolution compared to HT‐CO_2_RR. Thus, Co and Fe cations in the perovskite bulk structure can be reduced to metals with atomic ratios near that in the parent LSC_0.2_F_0.8_ (Figures S18–S20, Tables S2 and S3, Supporting Information). When the applied potential reaches ≈2.25 V, the integrated area and fraction of metallic Fe decline to half that at OCV (Figure 2c; Figure S29b,d, Supporting Information). The NAP‐XPS studies provide two key observations: (I) coke‐free HT‐CO_2_RR suppresses Co and Fe exsolution compared to coke formation (Figure 2b‐d; Figure S29, Supporting Information); (II) coke removal takes place in parallel with the reoxidation of Fe(0) to Fe(III) (Figure 2c; Figure S29d, Supporting Information). Taking these observations together, we concluded that the adsorption of CO_2_* at the oxygen vacancy re‐oxidizes the exsolved CoFe alloy particles back to LSC_0.2_F_0.8_. This argument was supported by the literature finding that CO_2_ re‐oxidizes the exsolved metals from the parent perovskites.^[^ ^19^ ^]^ As the number of CoFe particles decreases, the coke resistance is enhanced; at the same time, the density of metal‐oxide interfaces also decreases, ruling out its role in determining the coke resistance of LSC_1‐x_F_x_. Moreover, the quasi‐in‐situ Raman analysis for cells operated at 650 °C and a 50‐sccm inlet gas flow rate, where the thermodynamic coke formation threshold is 0.43 atm, agrees with the C 1s XPS at 700 °C under stationary CO_2_/CO atmosphere (Figure 2b) and the coke resistance seen on different LSC_1‐x_F_x_ at 800 °C and 60 sccm (Figure 1e). This confirms the universality of the decisive impact of the B‐site Fe vs. Co ratio on coke resistance across different operation conditions. Specifically, coke formation on LSC_0.2_F_0.8_ and LSF occurs at 0.4 atm, close to the thermodynamic threshold. In stark contrast, the D and G bands of deposited carbon were found at 0.15 atm outlet P_CO_ on LSC (Figure 2e), implying a much earlier onset of coking. These observations link coke resistance on LSC_1‐x_F_x_ perovskite oxides to their Fe stoichiometric ratio at the B site.

### 2.3 Theoretical Calculations

**Figure 3:**
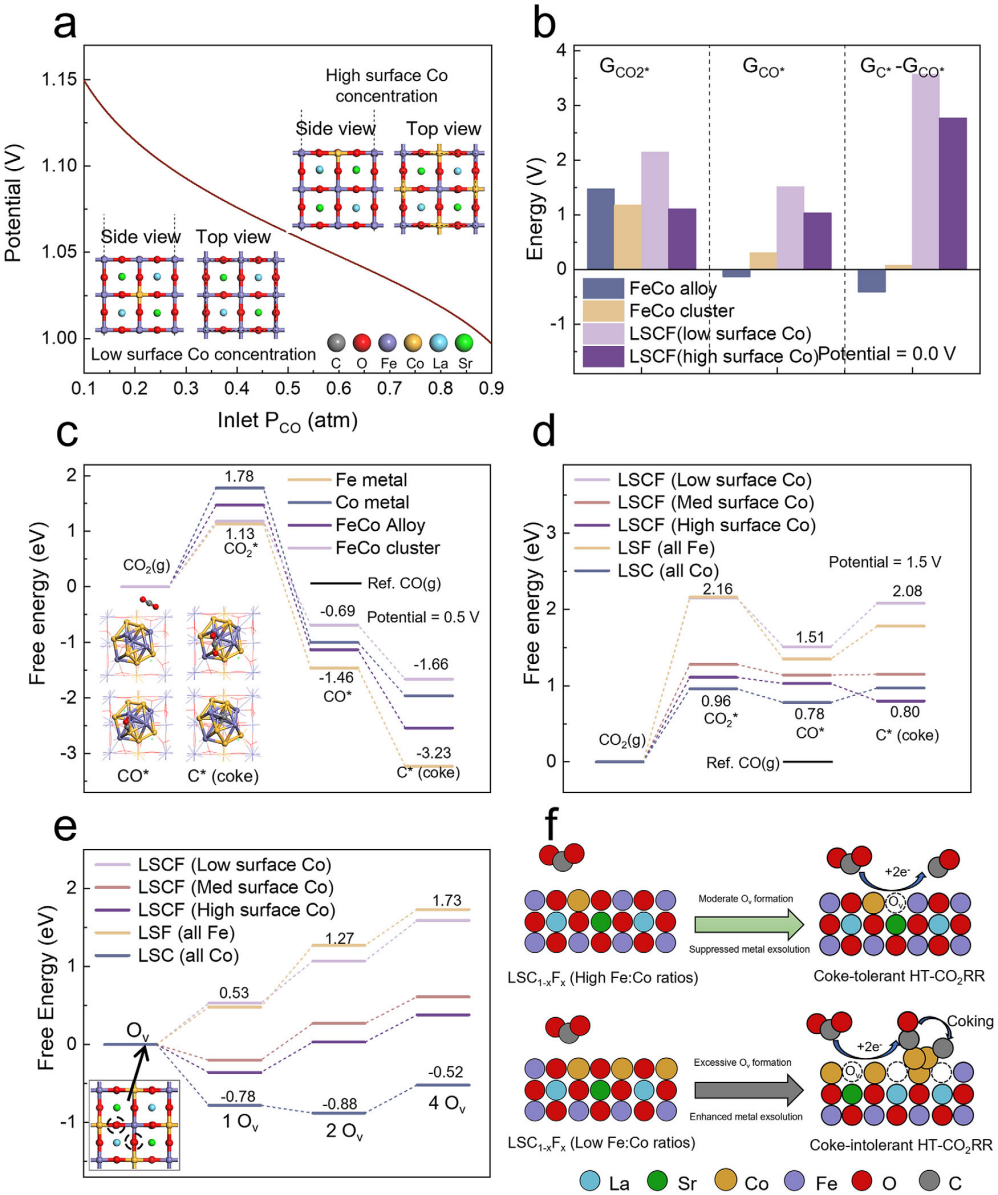
DFT calculations. a) The most stable LSCF surfaces at different inlet CO partial pressure and cell potential. LSCF with high surface Co concentration is the most stable at any position above the brown line, and vice versa. The sum of partial pressure of CO and CO_2_ is 1. b) The free energies of CO_2_ and CO adsorption (G_CO2_* and G_CO_*), and the free energy change of CO* → C* (G_C_* – G_CO_*) on different surfaces. c) Free energy profiles of HT‐CO_2_RR on Fe/Co metal, alloy, and cluster at 0.5 V. Inserts are the structures of CO_2_ g), CO_2_*, CO*, and C* (coke) on a Fe/Co cluster over LSCF surface. The ball‐and‐stick style models represent the clusters, while the line‐style models underneath represent the LSCF slabs. d) Free energy profiles of HT‐CO_2_RR on LSC/LSF and LSCF with low, medium, and high surface Co concentrations at 1.5 V. e) Free energy profiles of O_v_ formation on LSCF with low, medium, and high surface Co concentrations. f) An illustration of HT‐CO_2_RR, O_v_ formation capacity, and coke resistance mechanism on LSCF surface. More details and structures can be found in Note S1 (Supporting Information).

We sought to study further coke formation and removal in in situ NAP‐XPS and quasi‐in‐situ Raman using density functional theory (DFT) calculations. Given the exsolved B‐site metal sockets on LSCF(100) surfaces and the high symmetry of the LSCF cell (Figures S24 and S31, Supporting Information), we utilized the LSCF(001) facet for the calculations. Structural stability calculations indicate that, at low cathodic potentials and inlet P_CO_, the lanthanum strontium cobalt ferrite (LSCF) surface is Fe‐rich (**Figure** **3**a). Along with the applied potential and the inlet P_CO_ increase, Co atoms in the bulk of the LSCF slab migrate to the surface, and the surface Co concentration rises from low to high (Figure 3a; Figure S32 and Note S1, Supporting Information). This agrees with our observation that exsolved metal particles have low Fe:Co ratios under HT‐CO_2_RR without coking. This process also reduces the Fe oxidation state (from +3 to +2.7) and decreases O_v_ formation energy from 0.53 to −0.36 eV. The result is in line with the prior study that surface Co promotes O_v_ formation.^[^ ^28^ ^]^

The calculated HT‐CO_2_RR free energy profiles on pure Ni, Co, Fe metals, Co/Fe alloy, and Co/Fe clusters show much lower energy changes for the *CO‐to‐*C step (G_C_* – G_CO_*) and stronger CO adsorption energy (G_CO_*) than the perovskite surfaces (Figure 3b,c; Figure S33 and Table S8, Supporting Information). This aligns with the experimental results and confirms that the exsolved metal/alloy particles are active sites for coking (Figure 2b‐d; Figures S18–S20, and S29, Supporting Information). When Co atoms migrate to the surface, O_v_ formation energy reduces (from 0.52 to −0.36 eV). The following increase in O_v_ concentration on Co‐rich perovskite surfaces (Figure S33, Supporting Information) strengthens the adsorption of CO_2_* (from 2.15 to 1.11 eV) (Figure 3b,d). Consequently, CO_2_* dissociation (CO_2_* → CO* + O^2–^) injects O atoms into O_v_, which oxides the low‐valence Co and Fe, including the exsolved CoFe particles, back to higher valence states (e.g., Fe(III)) (Figure S35, Supporting Information). With suppressed CoFe alloy formation on surfaces, coke formation turns thermodynamically unfavorable at 0 and 1.5 V (Figure 3b,d), and coke removal (i.e., the C*‐to‐CO* process) is promoted conversely. Due to the weaker CO* adsorption on LSCF (G_CO_* is 1.03–1.51 eV) than metals (Figure 3d; Note S1, Supporting Information), CO* is prone to desorb rather than dissociate into *C and O^2–^ on LSCF.

Figure 3e compiles the energy profiles of O_v_ formation on LSC, LSF, and LSCF with different surface Co concentrations. LSC is the most favorable surface for O_v_ formation among these surface structures, showing downhill energy changes from the first (O_v_1, −0.78 eV) to the fourth O_v_ (O_v_4, 0.26 eV). Therefore, LSC is expected to have excessive O_v_ concentration and more Co exsolution under HT‐CO_2_RR conditions, a hypothesis confirmed by the SEM images of different LSC_1‐x_F_x_ electrodes after HT‐CO_2_RR (Figure S36, Supporting Information). With more B‐site cations being reduced to metal nanoparticles on perovskite surfaces, the density of coke‐formation active sites (i.e., the exsolved metals) increases (Figure 3c) despite pristine LSC, LSF, and LSCF possessing undesired energy changes for coking (Figure 3d). In contradistinction, both O_v_1 (ΔG = −0.36−0.53 eV) and O_v_2 (ΔG = 0.39−0.79 eV) are less thermodynamically favorable on LSF and LSCF than LSC, resulting in suppressed B‐site‐metal exsolution seen in Figure S36 (Supporting Information) and thus strong coke resistance (Figure 3f). It should be noted that the Fe vs. Co ratio reflects the B‐site composition at the surface of the parent LSC_0.2_F_0.8_ perovskites under conditions with and without coke formation. According to the calculation in Figure 3c,d, all metals and alloys favor coke deposition, and all perovskite surfaces, regardless of the Fe vs Co ratio, have high coke resistance. Under CO_2_ electrolysis conditions, the real catalysts comprise parent LSC_1‐x_F_x_ and the exsolved B‐site metals/alloys. What is determined by the surface Fe vs. Co ratio is the reaction free energy changes for forming more Ov and exsolved B‐site metal or alloy particles. This change is the most negative in the case of LSC, as it possesses the highest Co ratio on surfaces. With the increase of the surface Fe fraction, Ov formation and metal exsolution become less thermodynamically favorable (Figure 1e). As a result, fewer coke‐active centers are formed, and the coke resistance of the electrode increases. To further investigate the underlying mechanism, we calculated the charge density difference of oxygen atoms as in surface Co‐O‐Fe and Fe‐O‐Fe structures before and after oxygen vacancy (O_v_) formation (Figure S37, Supporting Information). The results reveal that oxygen atoms in Co─O─Fe bonds (1.12 e) gain more electrons compared to those in Fe─O─Fe bonds (0.97 e). Specifically, Co doping enhances electron transfer to oxygen, thereby facilitating the formation of O^2–^ and leading to the formation of O_v_ on the surface. This electronic redistribution weakens the metal‐oxygen bonds at Co sites, thermodynamically favoring O_v_ generation.

### 2.4 Long‐Term Performance and the Tandem System

**Figure 4:**
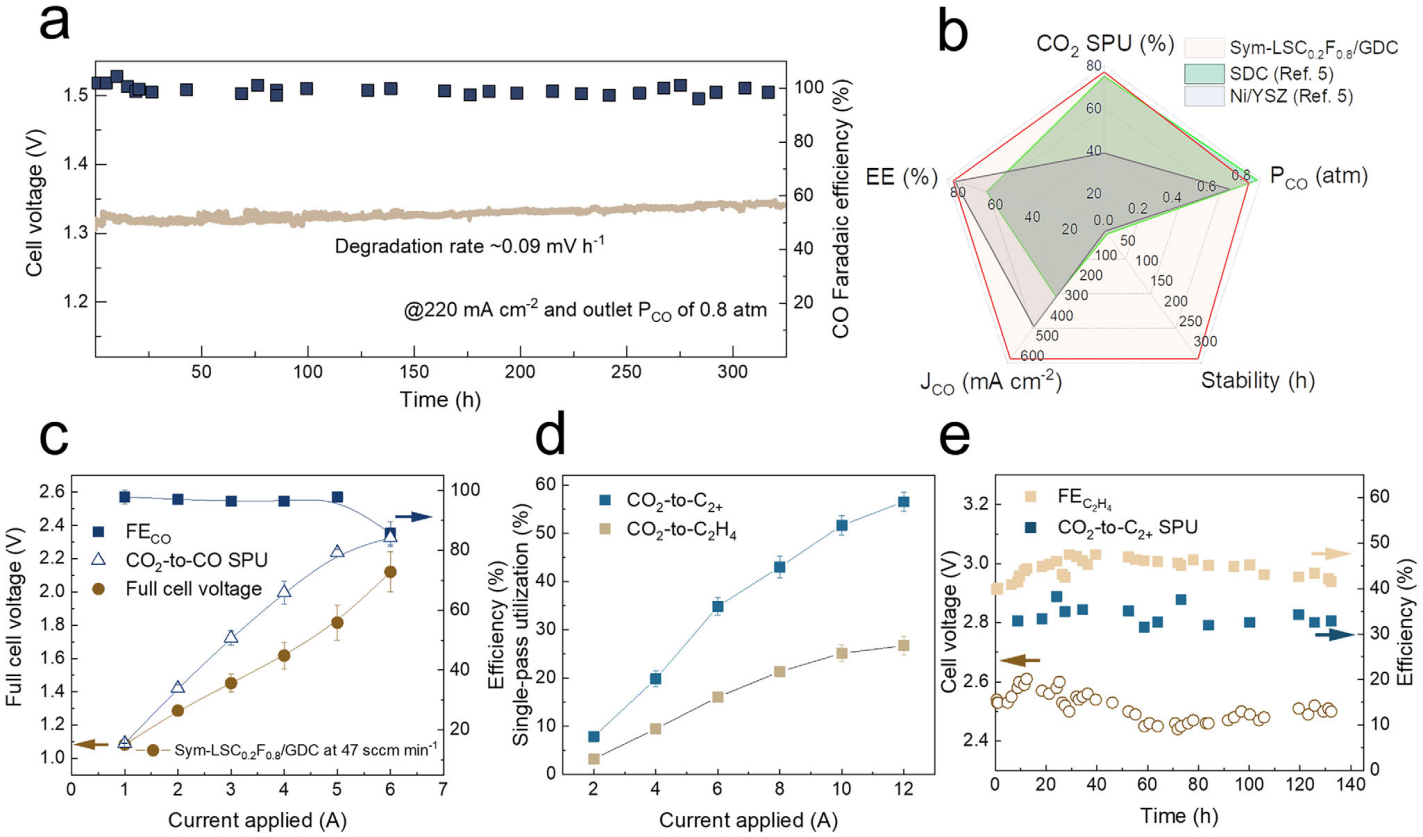
The long‐term HT‐CO_2_RR operation at 800 °C. a) A 320‐h stability test of HT‐CO_2_RR on the sym‐LSC_0.2_F_0.8_/GDC cell at 220 mA cm^−2^ and an outlet P_CO_ of 0.8 atm. b) Comparison of CO_2_ SPU, outlet P_CO_, stability, current density, and electrolysis EE (at>200 mA cm^−2^) achieved in the presented advance to the best‐prior reports. c) Cell voltage, CO FE, and CO_2_‐CO SPU of the 60 cm^2^ sym‐LSC_0.2_F_0.8_/GDC cells in a pure CO_2_ atmosphere. d) The CO_2_ SPU to C_2+_ products and C_2_H_4_ stand at different applied currents. e) Stability of the cell voltage, C_2_H_4_ FE, and CO_2_‐to‐C_2+_ SPU at 6 A. The total flow rates of CO_2_ and CO for these measurements are 50 sccm. The tandem system uses membrane electrode assembly (MEA) with a 100 cm^2^ active area.

We characterized the stable operation of catalysts in CO_2_ electrolysis above 200 mA cm^−2^. Over a 320‐h test at 220 mA cm^−2^, at an inlet P_CO_ of 0.60 atm (**Figure** **4**a), we observed CO Faradaic efficiency of ≈100% and an electrolysis EE of ≈74% (equivalent to a heat‐included EE of ≈70%) at an outlet P_CO_ of 0.80 atm, and cell degradation rate of ≈0.09 mV h^−1^ (equivalent to 0.3% h^−1^). This represents higher stability with improved coke resistance compared to previous reports (Figure 4b; Table S9, Supporting Information).^[^ ^5^, ^16^, ^17^, ^18^, ^19^, ^20^, ^21^, ^22^, ^23^ ^]^

We developed a tandem CO_2_ electro‐upgrading system by connecting the sym‐LSC_0.2_F_0.8_/GDC cell and a 100 cm^2^ CO electroreduction membrane electrode assembly (MEA) in series, bringing the entire process into a stage of multi‐carbon (C_2+_) product synthesis (Figure S38, Supporting Information). Cu_2_O‐derived Cu gas diffusion electrodes were employed to convert the CO_2_‐synthesized CO (Figure 4c) to ethylene, ethanol, acetaldehyde, acetate, and n‐propanol (Figure S39, Supporting Information).^[^ ^29^ ^]^ We kept the outlet P_CO_ and current density for the SOEC at 0.8 atm and 220 mA cm^−2^ and ran the MEA at different currents. We reported an 80% Faradaic efficiency for C_2+_ products at 6 A (Figure S40, Supporting Information) and a CO_2_‐to‐C_2+_ SPU of 57% ± 2% at 12 A (Figure 4d). The corresponding CO_2_‐to‐C_2_H_4_ conversion is ≈27 ± 1%, nearly three times as high as the previous report.^[^ ^4^ ^]^ The CO_2_‐to‐C_2+_ SPU was sustained near‐constant at 35% and 50% during 130‐ and 50‐h electrolysis at 6 A and 10 A for the MEA reactor, respectively (Figure 4e; Figure S40, Supporting Information).

## 3 Conclusion

In summary, this work links the coke resistance and B‐site Fe:Co ratio via the following chemical picture: higher Fe stoichiometric ratios at the B site of LSC_1‐x_F_x_ avoid the formation of excessive surface oxygen vacancies during HT‐CO_2_RR and suppress the exsolution of coking‐active metallic particles. This dynamic change of surface composition, including both the parent perovskites and exsolved metals, in response to the operation condition and the surface composition‐coke resistance correlation were absent in previous publications and proved experimentally and theoretically in our work. As a result, LSC_0.2_F_0.8_‐based symmetric SOECs provide an outlet P_CO_ limit of 0.86 ± 0.02 atm and a CO_2_ SPU of 75 ± 2% at 800 °C, equivalent to a ≈1.4× increase in coke resistance compared to the commercial Ni/YSZ cell. We demonstrated, using the sym‐LSC_0.2_F_0.8_/GDC cells, 320‐h operation producing downstream P_CO_ of ≈0.8 atm at 220 mA cm^−2^ with an electrolysis EE of 74%, a heat‐included EE of 70%, and a degradation rate of ≈0.09 mV h^−1^. We built a tandem CO_2_‐CO‐C_2+_ reactor system, upgrading CO_2_ to C_2+_ products at an overall Faradaic efficiency of ≈80%, CO_2_‐to‐C_2+_ SPU of 57 ± 2%, and stability of 130 h at 6 A. The mechanistic findings offered by this work deepen the understanding of the HT‐CO_2_RR and coke formation dynamics, and the performance advances achieved underscore the potential of CO_2_ electrolysis as a carbon‐neutral chemical production technology.

## Conflict of Interest

The authors declare no conflict of interest.

## Supporting information



Supporting Information

## Data Availability

The data that support the findings of this study are available in the supplementary material of this article.

## References

[1] R. Küngas , J. Electrochem. Soc. 2020, 167, 044508.

[2] X. Lv , M. Chen , Z. Xie , L. Qian , L. Zhang , G. Zheng , Chin. J. Catal. 2022, 43, 92.

[3] T. Wang , G. Han , Z. Wang , Y. Wang , Chin. J. Catal. 2022, 43, 2938.

[4] A. Ozden , Y. Wang , F. Li , M. Luo , J. Sisler , A. Thevenon , A. Rosas‐Hernández , T. Burdyny , Y. Lum , H. Yadegari , T. Agapie , J. C. Peters , E. H. Sargent , D. Sinton , Joule 2021, 5, 706.

[5] T. L. Skafte , Z. Guan , M. L. Machala , C. B. Gopal , M. Monti , L. Martinez , E. Stamate , S. Sanna , J. A. Garrido Torres , E. J. Crumlin , M. García‐Melchor , M. Bajdich , W. C. Chueh , C. Graves , Nat. Energy 2019, 4, 846.

[6] T. L. Skafte , C. Graves , P. Blennow , J. Hjelm , ECS Trans. 2015, 68, 3429.

[7] M. Navasa , H. L. Frandsen , T. L. Skafte , B. Sundén , C. Graves , J. Power Sources 2018, 394, 102.

[8] W. Li , Y. Shi , Y. Luo , Y. Wang , N. Cai , J. Power Sources 2015, 276, 26.

[9] T. L. Skafte , P. Blennow , J. Hjelm , C. Graves , J. Power Sources 2018, 373, 54.

[10] N. Li , L. Wang , M. Wang , X. Ban , C. Chen , Z. Zhan , J. Power Sources 2022, 518, 230787.

[11] A. K. Clausen , M. L. Traulsen , P. V. Hendriksen , X. Sun , ECS Trans. 2021, 103, 1945.

[12] V. Duboviks , R. C. Maher , M. Kishimoto , L. F. Cohen , N. P. Brandon , G. J. Offer , Phys. Chem. Chem. Phys. 2014, 16, 13063. 24871047 10.1039/c4cp01503g

[13] H. Shin , K. U. Hansen , F. Jiao , Nat. Sustain. 2021, 4, 911.

[14] T. Moore , D. I. Oyarzun , W. Li , T. Y. Lin , M. Goldman , A. A. Wong , S. A. Jaffer , A. Sarkar , S. E. Baker , E. B. Duoss , C. Hahn , Joule 2023, 7, 782.

[15] M. Jouny , W. Luc , F. Jiao , Ind. Eng. Chem. Res. 2018, 57, 2165.

[16] H. Lv , T. Liu , X. Zhang , Y. Song , H. Matsumoto , N. Ta , C. Zeng , G. Wang , X. Bao , Angew. Chem., Int. Ed. 2020, 59, 15968. 10.1002/anie.202006536 32452143

[17] S. Lee , M. Kim , K. T. Lee , J. T. S. Irvine , T. H. Shin , Adv. Energy Mater. 2021, 11, 2100339.

[18] H. Lv , L. Lin , X. Zhang , R. Li , Y. Song , H. Matsumoto , N. Ta , C. Zeng , Q. Fu , G. Wang , X. Bao , Nat. Commun. 2021, 12, 5665. 34580312 10.1038/s41467-021-26001-8 PMC8476569

[19] Y. Shen , T. Liu , R. Li , H. Lv , N. Ta , X. Zhang , Y. Song , Q. Liu , W. Feng , G. Wang , X. Bao , Natl. Sci. Rev. 2023, 10, nwad078. 37565207 10.1093/nsr/nwad078 PMC10411681

[20] F. He , M. Hou , F. Zhu , D. Liu , H. Zhang , F. Yu , Y. Zhou , Y. Ding , M. Liu , Y. Chen , Adv. Energy Mater. 2022, 12, 2202175.

[21] Z. Wang , T. Tan , K. Du , Q. Zhang , M. Liu , C. Yang , Adv. Mater. 2023, 36, 2312119. 10.1002/adma.202312119 38088211

[22] T. Tan , Z. Wang , M. Qin , W. Zhong , J. Hu , C. Yang , M. In Liu , Adv. Funct. Mater. 2022, 32, 2202878.

[23] H. Lv , L. Lin , X. Zhang , Y. Song , H. Matsumoto , C. Zeng , N. Ta , W. Liu , D. Gao , G. Wang , X. In Bao , Adv. Mater. 2020, 32, 1906193. 10.1002/adma.201906193 31894628

[24] D. Neagu , T. S. Oh , D. N. Miller , H. Menard , S. M. Bukhari , S. R. Gamble , R. J. Gorte , J. M. Vohs , J. T. S. Irvine , Nat. Commun. 2015, 6, 8120. 26360910 10.1038/ncomms9120 PMC4579408

[25] S. Wang , M. Katsuki , M. Dokiya , T. Hashimoto , Solid State Ion. 2003, 159, 71.

[26] P. Hjalmarsson , X. Sun , Y.‐L. Liu , M. Chen , J. Power Sources 2013, 223, 349.

[27] Y. Tao , S. D. Ebbesen , M. B. Mogensen , J. Electrochem. Soc. 2014, 161, F337.

[28] A. M. Ritzmann , J. M. Dieterich , E. A. Carter , Phys. Chem. Chem. Phys. 2016, 18, 12260. 27079696 10.1039/c6cp01720g

[29] C.‐T. Dinh , T. Burdyny , M. G. Kibria , A. Seifitokaldani , C. M. Gabardo , F. P. G. De Arquer , A. Kiani , J. P. Edwards , L. De , P. Bushuyev , Science 2018, 360, 783. 29773749 10.1126/science.aas9100

