# Supplementary material for: B‐Site‐Metal‐Mediated Coke‐Resistant CO2 Electrolysis on Perovskite Surfaces
Source: Adv Sci (Weinh). 2025 May 21;12(29):e03970. doi: 10.1002/advs.202503970 (PMC12362793; doi:10.1002/advs.202503970)
Supplement: Supplementary file 1 — Supporting Information [file ADVS-12-e03970-s001.pdf]

ADVANCED SCIENCE
Open Access

# Supporting Information

for *Adv. Sci.*, DOI 10.1002/advs.202503970

B-Site-Metal-Mediated Coke-Resistant $CO_2$ Electrolysis on Perovskite Surfaces

*Tongbao Wang, Yu Mao, Pengfei Ou, Zhijie Wang, Yifan Li, Hao Li, Binbin Pan, Ximeng Lv, Yanguang Li, Gengfeng Zheng, Chengzhi Guan, Yi Cui, Ziyun Wang* and Yuhang Wang**

Supporting Information

**B-Site-Metal-Mediated Coke-Resistant $CO_2$ Electrolysis on Perovskite Surfaces**

*Tongbao Wang+[a][b], Yu Mao+[c], Pengfei Ou+[d], Zhijie Wang+[e], Yifan Li[f], Hao Li[f], Binbin Pan[a][b], Ximeng Lv[g], Yanguang Li[a][b], Gengfeng Zheng[g], Chengzhi Guan[e], Yi Cui[f], Ziyun Wang*[c], Yuhang Wang*[a][b]*

## Materials and Methods

### The cell fabrication

Unless otherwise noted, all reagents were from Shanghai Aladdin Bio-Chem Technology Co., Ltd, and used without further purification. $LSC_{0.2}F_{0.8}$ catalysts were purchased from Ningbo SOFCMAN Energy Technology Co., Ltd., China. Other $LSC_{1-x}F_x$ were synthesized via a sol-gel method. In a typical procedure, stoichiometric amounts of high-purity $La(NO_3)_3 \cdot 6H_2O$, $Sr(NO_3)_2$, $Co(NO_3)_2 \cdot 6H_2O$, and $Fe(NO_3)_3 \cdot 9H_2O$ were dissolved in deionized (DI) water. Subsequently, citric acid (CA) and ethylenediaminetetraacetic acid (EDTA) were added to the solution in a molar ratio of metal ions:CA:EDTA = 1:1.5:1. The pH of the resulting solution was adjusted to approximately 7 using ammonia. The mixture was continuously heated and stirred at 200°C to form a gel. This gel was then subjected to a preliminary treatment at 250°C for over 2 hours to obtain the precursors. The resulting precursors were calcined at 950°C for 6 hours to produce the $La_{0.6}Sr_{0.4}Co_{1-x}Fe_xO_{3-\delta}$ ($LSC_{1-x}F_x$, where x = 0, 0.2, 0.5, 0.8, and 1) powder.

For cell fabrication, GDC powder and SSZ electrolyte sheets were purchased from Ningbo SOFCMAN Energy Technology Co., Ltd., China. To prevent the reaction between $LSC_{1-x}F_x$ and SSZ, a GDC buffer layer was first coated onto the SSZ sheet by screen printing the ink consisting of the GDC powder, polyvinyl butyral (PVB) binder, dioctyl phthalate (DOP) dispersant, 1-butanol, and terpineol and firing at 1350°C. Then, the $LSC_{1-x}F_x$ catalyst was mixed with GDC and fired at 1100°C. The obtained powders were then mixed with PVB binder, DOP dispersant, polymethylmethacrylate (PMMA) pore former (3 μm diameter, Suzhou Soken Chemical Co., Ltd), and 1-butanol in terpineol by ball milling to form a homogenous ink. The electrode ink was screen printed onto the GDC-coated SSZ electrolyte sheet with an effective area of ~5 $cm^2$ or 60 $cm^2$ for the cathode. The anode was $LSC_{0.2}F_{0.8}$/GDC. After printing one electrode (i.e., the cathode or anode), the as-made half cell was fired at 1150°C. The same procedure was then repeated for the other electrode. The structure of the fabricated cell is $LSC_{1-x}F_x$-GDC|GDC|SSZ|GDC|$LSC_{0.2}F_{0.8}$-GDC. The comm-Ni/YSZ cells (cathode porosity 25% before reduction) were purchased from Ningbo SOFCMAN Energy Technology Co., Ltd., China.

### Material characterization

XRD studies were performed using a PANalytical Empyrean diffractometer (Cu Kα radiation, λ=1.54 Å). SEM images were obtained on ZEISS G500. TEM measurements were carried out on TALOS 200X and JEOL JEM-2100F. EDS elemental maps were also obtained using EDS built-in TALOS 200X and Oxford Instruments X-Max 80T. Raman spectroscopy measurements were carried out on a LabRAM HR 800 Raman spectrometer (Horiba). The porosity result was determined by analyzing an image obtained through Scanning Electron Microscopy (SEM). This analysis utilized the “Trainable Weka Segmentation” function, which is located within the “Segmentation” option under the “Plugins” dropdown menu in the ImageJ software. The oxygen vacancy concentration was determined by analyzing weight loss using thermogravimetric analysis (TGA) performed with a Mettler TGA instrument (Switzerland) in air.

**Electrochemical $CO_2$-to-CO reduction measurements**

The high-temperature $CO_2$ electrolysis was operated using an all-in-one test system from Ningbo SOFCMAN Energy Technology Co., Ltd., China. The measurements were performed under ambient pressure at 800°C. Different $CO/CO_2$ mixtures were fed into the cathode at a constant 50 standard cubic centimeters (sccm) flow rate, and the anode was exposed to air (100 sccm). Besides, the stability test was fixed at a 60 sccm flow rate, with inlet $P_{CO}$ at 0.65 atm. For testing the comm-Ni/YSZ cell, 5% $H_2$ in Ar (100 sccm) was first injected into the cathode to reduce NiO to Ni. When a stable OCV of ~1.1 V was reached, the cathodic feedstock was switched to $CO/CO_2$ mixtures.

The products were analyzed using online gas chromatography (GC 2060) equipped with an autosampler. The measurements at different voltages were conducted using Metrohm Autolab potentiostat/galvanostat (PGSTAT 204N) equipped with a 10 A booster.

**The $CO_2$-CO-$C_{2+}$ tandem reactor**

The tandem reactor consists of a SOEC setup and a zero-gap CORR electrolyzer (100 $cm^2$, Shanghai Keqi). The two reactors are connected via a $CO_2$ separation unit using calcium lime. The SOEC-produced CO is purified and cooled down to 25°C in the separation unit before being supplied to the CORR electrolyzer.

For the CORR test, the $Cu_2O$ precatalyst was prepared by drying the reaction product of KOH (Shanghai Aladdin Bio-Chem Technology Co., Ltd) and CuCl (Sigma Aldrich) dissolved in hydrochloric acid (Shanghai Lingfeng Chemical Reagent Co. Ltd) solution. The $Cu_2O$ precatalyst was airbrushed onto a gas diffusion layer (AvCarb MB30). Carbon black/Nafion ionomer (10 µL, Sigma Aldrich, 5 wt. % in lower aliphatic alcohols and water) and graphite/Nafion (10 µL) layers were sequentially deposited on the Cu electrode by airbrushing. 1 M KOH (Sigma Aldrich) was used as the anolyte. Ni foam-supported NiFe hydroxides were the anode catalyst for water oxidation and were prepared by electrochemical deposition of 3 mM $Ni(NO_3)_2 \bullet 6H_2O$ (Sigma Aldrich) and 3 mM $Fe(NO_3)_3 \bullet 9H_2O$ (Sigma Aldrich) at -1.0 V vs. Ag/AgCl for 5 min, according to a previously established method.[1] An anion-exchange membrane (Sustainion X37-50 Grade 60) was placed between the cathode and anode. The CORR measurements were performed at currents of 2 to 12 A.

Gas-phase products were detected by online gas chromatography (GC 2060). Liquid products were analyzed using high-performance liquid chromatography (Thermo VanquishCore). The measurements at different voltages were conducted using Metrohm Autolab potentiostat/galvanostat (PGSTAT 204N) equipped with a 10 A booster.

**Faradaic efficiency (FE) and energy efficiency (EE) calculations**

FEs for different gas products were calculated using the following equation:

$$\mathrm{FE} = \frac{\mathrm{F} \times \mathrm{n_a} \times \mathrm{V_{gas}} \times \mathrm{c_a}}{\mathrm{i} \times \mathrm{V_m}} \tag{1}$$

Where $F$ stands for the Faraday constant, $n_a$ stands for the number of electron transfers needed for producing 1 mol of product a, $V_{gas}$ stands for the flow rate of the supplied $CO_2$, $c_a$ stands for the detected concentration of product a, i stands for the overall operating current, and $V_m$ is the unit molar volume of gases at 298.15 K (24.5 L $mol^{-1}$).

For liquid products, FEs were calculated using the equation below:

$$FE = m_b \times \frac{F \times n_b}{i \times t} \tag{2}$$

Where $F$ stands for the Faraday constant, $n_b$ stands for the number of electron transfers needed for producing 1 mol of product b, $m_b$ stands for the mole of product b, i is the overall operating current, and $t$ corresponds to the electrolysis duration.

The electrolysis energy efficiency of the SOEC was calculated using the equation below:

$$\text{Electrolysis EE} = \frac{E^0_{CO} \times FE_{CO}}{\text{Applied voltage}} \tag{3}$$

Where $E^0_{CO}$ represents the thermodynamic cell potential for $CO_2$ reduction to CO (0.97 V at 800°C). $FE_{CO}$ represents the FE for CO.

When heat is involved and is not free, the heat-included energy efficiency of the SOEC was calculated using the equation below:[3]

$$\begin{aligned}\text{Heat} - \text{included EE} &= \frac{\Delta H_{CO2-to-CO} \times n_{CO,measured} \times FE_{CO}}{V \times I + T\Delta S n_{CO,measured} \times FE_{CO} + \left(V - \frac{\Delta G}{nF}\right) I} \times 100\% \\ &= \frac{\Delta H_{CO2-to-CO} \times n_{CO,measured} \times FE_{CO}}{V \times I + T\Delta S n_{CO,mesured} \times FE_{CO} + (V - E^0_{CO}) I} \times 100\%\end{aligned} \tag{4}$$

Where $\Delta H_{CO2\text{-}to\text{-}CO}$ represents the reaction enthalpy for $CO_2$ reduction. $n_{CO,measured}$ is the measured CO production rate (mol $s^{-1}$). V and I is the voltage (V) and current (A) applied. T represents the operating temperature (K). $\Delta S$ is the reaction entropy for $CO_2$ reduction at operating temperature.

**In-situ NAP-XPS measurements**

For the model cell used for in-situ NAP-XPS characterization, the $LSC_{0.2}F_{0.8}$-GDC electrode ink does not contain PMMA pore former. A gold wire grid (~20 µm diameter) was placed on top of the printed cathode before co-firing the cell at 1050°C, serving as the current collector. Ag paste was applied to the edge of the cathode to hold the cathode current collector in place. Ag paste (SPI paint) was also used to connect the anode with a gold wire current connector. The effective area of the cell is ~0.5 $cm^2$. The sample holder has a sapphire wafer to ensure a uniform temperature distribution on the sample surface. A vermiculite O-ring was positioned between the sample and holder to avoid the short circuit (Figure S27a). The edge of the cell was sealed using a high-temperature silicate sealant (Figure S27b).

The in-situ NAP-XPS measurements were performed using a SPECS NAP-XPS instrument connected to a glovebox. The photo source was a monochromatic Al-Kα X-ray radiation (1486.6 eV). The transmitting power used is 14 kV and 40 W. The laser power for heating the cell is 25 W, leading to a sample temperature of ~700°C. The loaded cell was tested at OCV, and cell voltages of 5 V, 7 V, and 9 V without iR correction, respectively. The spectra were collected after 5 min of stable operation in an elemental order of Au, C, O, Fe, and Co. All acquired spectra were calibrated using the Au 4f spectrum (centered at 84 eV).

The fitted peak positions for all species were fixed at the same position, respectively, with a deviation of < 0.2 eV. The binding energy intervals were set to eV for 282-288 eV for C 1s, 525-535 eV for O 1s, 703-728 eV for Fe 2p, and 773-794 eV for Co 2p. The half-peak width of the same valence state for the same element was kept identical. The integrated area of Co(0) and Co(III) is the area of Co(0) 2p 3/2 and Co(III) 2p 3/2 spectrum divided by an atomic sensitivity factor of 3.59,[3] respectively. Fe (0), Fe (III), O, and C spectra are processed similarly. The atomic sensitivity factor is 2.957 for Fe, 0.711 for O, and 0.296 for C.[3]

**Quasi-in-situ Raman spectroscopy studies**

The in-situ reaction equipment (HT-Raman-800B) was manufactured by Beijing Scistar Technology Co, Ltd. For the model cell used for quasi-in-situ Raman characterization, the $LSC_{1-x}F_x$ (x = 0, 0.2, and 1) electrode ink does not contain PMMA pore former. The fabricated cell was placed in a quartz tube and sealed with a silicate binder. Silver wire was employed as both the anode and cathode current collectors, secured with cured silver paste. The assembly was then placed into an in-situ reaction device, where it was heated to 650°C at a temperature ramping rate of 5°C per hour. Upon reaching 650°C, a gas flow meter was utilized to detect any discrepancies in flow rates between the inlet and outlet gases, thereby identifying potential leaks. Subsequently, currents of 10 mA, 30 mA, 50 mA, 100 mA, and 150 mA were applied sequentially under conditions. After the testing, the catalyst layer bonded to the silver paste was removed by peeling it off after a rapid cooldown in the reaction atmosphere. Carbon deposition was then examined using a laser with a wavelength of 532 nm.

**DFT calculations**

All calculations in the paper were spin-polarized and carried out with the Perdew–Burke–Ernzerhof (PBE)[4] functional in Vienna ab initio simulation package (VASP).[5,6] The D3 correction method[7] was employed to include van der Waals (vdW) interactions. The project-augmented wave (PAW) method was used to represent the core–valence interaction.[8,9] For the calculations of total energy, a cut-off energy of 500 eV was set for plane wave basis sets to expand the valence electronic states, and the converging criteria of the force on each relaxed atom below 0.05 eV/Å were used for structural optimizations. To describe Fe and Co, the DFT+U method is applied where the on-site coulomb correction was set on 3d orbitals of Fe and Co with an effective U value of 4.0 and 3.3 eV, respectively (as suggested in other theoretical works[10, 11]). All structures were assumed to be in a ferromagnetic (FM) state at the start of the calculation.[12, 13]

In terms of modeling (Figure S32), because of the high symmetry of the LSCF cell, 001,010, and 100 are computationally fully equivalent. A unit cell of $La_4Sr_4Co_2Fe_6O_{24}$ was taken as this is the most widely used model for $(La_{0.6}Sr_{0.4})_{0.95}Co_{0.2}Fe_{0.8}O_{3-\delta}$ system.[11, 14-17] The lattice parameters were a = 7.786 Å, b = 7.897 Å, c = 7.732 Å, α = β = γ = 90° after optimization with 4×4×4 Brillouin zone sampling. Then, a 2×2 three-layer 001 surface was modeled to further investigate the catalytic activity. The bottom layer was fixed, and the top two layers were fully relaxed during structural optimizations. The Brillouin zone was sampled 3×3×1, and a vacuum layer of 12 Å was applied to avoid lateral interactions. Structures are visualized using OVITO.[18] The transition states (TS) are determined by a constrained optimization scheme,[19, 20] which are verified until (i) all forces on atoms vanish; and (ii) the total energy reaches maximum along the reaction coordination but minimum with respect to the rest of the degrees of freedom.

The adsorption energy ($\Delta G_{ad}$) was defined as:

$$\Delta G_{ad} = G_{adsorbate+surface} - G_{adsorbate} - G_{surface} \quad (5)$$

Where $G_{surface}$, $G_{adsorbate}$, and $G_{adsorbate+surface}$ are the free energies of the surface, adsorbate in the gas phase, and adsorbate adsorbed on the surface, respectively. Standard formulas of statistical mechanics were used to calculate the thermodynamic correction, including zero-point-energy (ZPE), thermal energy, and entropy derived from partition functions.[21, 22] For surface adsorbates, only vibrational contribution was considered, including ZPE, vibrational thermal energy, and vibrational entropy. The ZPE correction is given by:

$$E_{ZPE} = \sum_i \frac{hv_i}{2} \quad (6)$$

Where h is Plank's constant and $v_i$ is vibrational frequency i which is calculated based on the harmonic oscillators approximation. The standard molar vibrational thermal energy contribution is calculated by:

$$U_{vib}^{o} = RT\sum_{i}\frac{\frac{hv_i}{k_B}}{e^{\frac{hv_i}{k_BT}}-1} \quad (7)$$

Where R is the gas constant, and $k_B$ is Boltzmann's constant. The standard molar vibrational entropy is calculated using the following expression:

$$S_{vib}^{o} = R\sum_{i}[\frac{\frac{hv_i}{k_BT}}{e^{\frac{hv_i}{k_BT}}-1} - \ln(1-e^{hv_i/k_BT})] \quad (8)$$

Therefore, the Gibbs free energies are obtained by:

$$G = E_{total} + E_{ZPE} + U^{o} - TS^{o} + RTlnP \quad (9)$$

where $E_{total}$ refers to the total energy obtained from DFT calculation and P is the partial pressure (neglected for adsorbed species). To avoid abnormal entropy contribution, frequencies less than 50 $cm^{-1}$ are set to 50 $cm^{-1}$. The temperature is set to the experimental condition of 800°C (1073.15 K), and the free energy of O* ($G_{O*}$) is calculated by $G_{CO_2*} - G_{CO*}$. When a cathodic potential (U) is applied, CO* free energy is decreased by 2 eU and C* free energy by 4 eU (as $CO_2$* + $2e^-$ → CO* + $O^{2-}$; CO* + $2e^-$ → C* + $O^{2-}$).

In Figure 3a of the main text, the most stable LSCF surfaces at different inlet CO partial pressure and cell potential were determined by comparing the Gibbs free of energy low, medium, and high surface Co concentration LSCF slab model (Figure S32). Surface adsorption of $CO_2$*, CO*, and C* were considered, and their stability and Gibbs energy changes upon adsorption were calculated using Equation 9 with the consideration of the Boudouard reaction and cathodic potential. According to the Boudouard reaction, $CO(g) \leftrightharpoons CO* \leftrightharpoons C* + CO_2(g)$, the free energy of C* is $G_{CO2(g)} - G_{CO(g)}$, and we assumed that the sum of the partial pressure of CO(g) and $CO_2$(g) is 1. For cathodic potential, CO* free energy is decreased by 2 eU and C* free energy by 4 eU based on the number of electrons transferred.

When there is no inlet CO(g) and no cathodic potential applied, the stability of low-surface-Co LSCF is more stable (-0.26 eV) than high-surface-Co LSCF. However, when Co migrates from the bulk to the surface, the adsorption of C* is significantly strengthened (-1.02 eV more stable than low-surface-Co $LSC_{1-x}F_x$). Therefore, with increased cell potential and inlet CO(g) partial pressure, the chemical potential of C* becomes lower, and high-surface-Co LSCF with C* eventually becomes more stable. The transition line was calculated and shown in Figure 3a (the brown line). High-surface-Co LSCF with C* will then complete the catalytic cycle, removing C* and producing CO(g).

In Figure S33, we calculated the free energy profile of $CO_2$ reduction to CO and coke formation on Ni, Fe, Co, and FeCo alloy metal surfaces at 800°C and 0 V / 0.5 V potential. All relevant structures are listed in Table S8.

In Figure S35, we calculated the transition state of $CO_2$* → CO* on the LSCF surface with high surface Co concentration. $CO_2$ bonds a surface Co upon adsorption with its O atom, and this O atom moves towards the surface vacancy to form the transition state. On the transition state, the O inserts into the O-vacancy, and the C bridges between the O and surface Fe. The transition state has a similar structure to the final state (LSCF-CO*); therefore, we assume the energy of the transition state is influenced by the cell potential as CO* (decreased by 2 eU). The forward activation energy ($E_a$) is 0.50 eV at 1.5 V potential, and the $E_a$ for the backward reaction is 0.37 eV. Both forward and backward $E_a$ are very small, and the reaction $CO_2$* → CO* readily takes place under operating conditions.

In Figure S35, we calculated the Bader charge of surface Fe and compared it with the Fe in bulk $Fe_2O_3$,[23] and found that the valence of Fe on the O-vacancy-free LSCF surface is ~3 and decreases to ~2.7 when an O-vacancy forms. With more O vacancies, the valence of surface metal will continue to decrease and finally become Fe/Co nanoparticle. The Fe/Co ratio in the produced nanoparticle depends on the surface Fe/Co ratio of LSCF. Therefore, by catalyzing $CO_2$* → CO* on LSCF with O-vacancies ($LSC_{1-x}F_x$-$O_v$), the low valence-state Fe is oxidized to Fe(III) by $CO_2$ as the O atoms in $CO_2$ refill the O-vacancies.

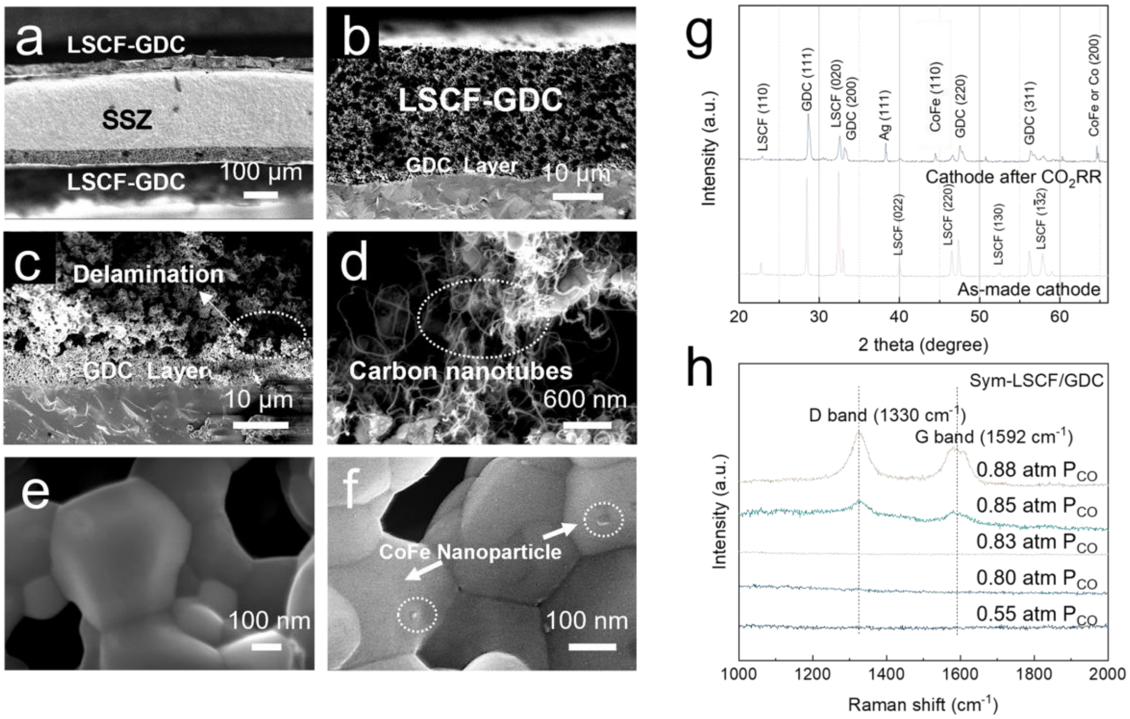


Figure S1. Ex-situ studies on the coke tolerance of the sym-$LSC_{0.2}F_{0.8}$/GDC cell. (a and b) Cross-section SEM images of the sym-$LSC_{0.2}F_{0.8}$/GDC cell before testing. (c and d) Cross-section SEM images of the sym- $LSC_{0.2}F_{0.8}$/GDC cell with coke formation after HT-$CO_2$RR. (e and f) SEM images of the $LSC_{0.2}F_{0.8}$-GDC electrode before HT-$CO_2$RR (e) and with CoFe exsolution after reaction (f). (g) The XRD analysis of the sym- $LSC_{0.2}F_{0.8}$/GDC cell before and after HT-$CO_2$RR. (h) The Raman spectra of sym- $LSC_{0.2}F_{0.8}$/GDC cells after testing at different outlet $P_{CO}$.

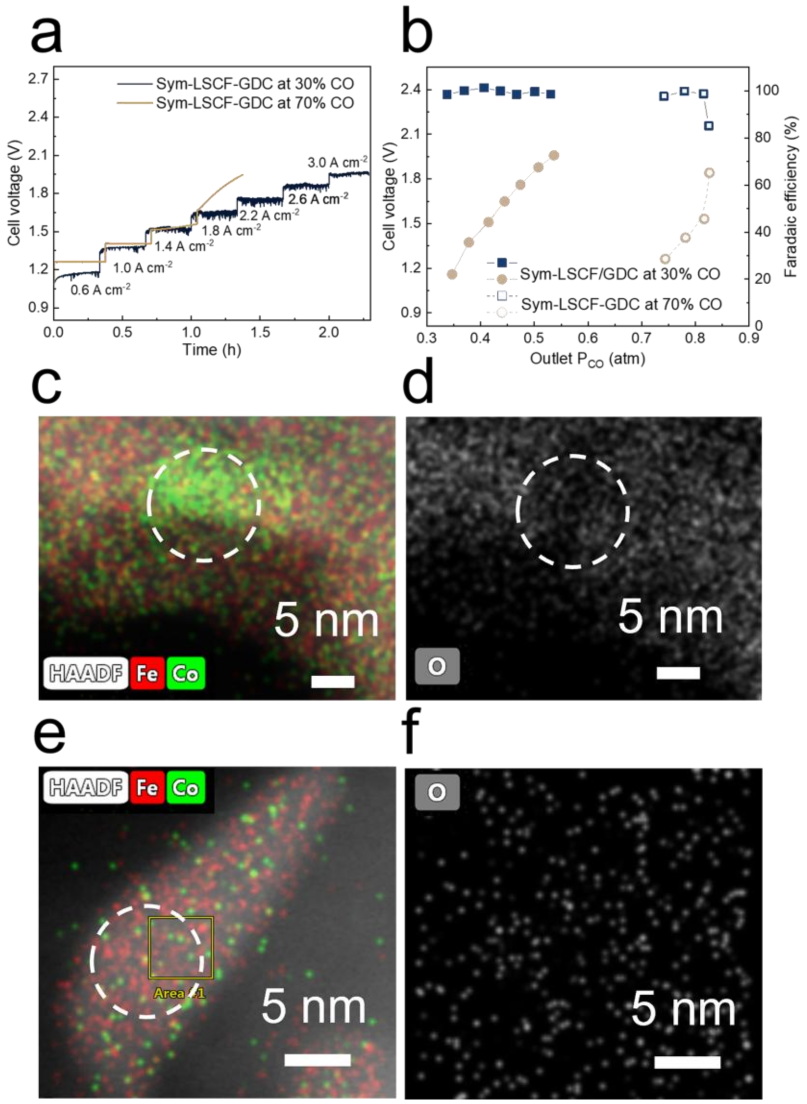


Figure S2. The performance of sym-$LSC_{0.2}F_{0.8}$/GDC cells with a 0.5 $cm^2$ effective area. (a) Voltage-time (V-t) profiles recorded at an initial current density of 600 mA $cm^{-2}$. Each subsequent potential step corresponds to an incremental increase of 400 mA $cm^{-2}$. (b) The cell voltages and CO FEs at different outlet $P_{CO}$. The inlet $P_{CO}$ was 0.3 atm inlet and 0.7 atm inlet, respectively, and the active area of each cell was 0.5 $cm^2$. (c, d) The high-angle annular dark-field (HAADF) image and corresponding EDS results of CoFe nanoparticles without coke formation. (e, f) The high-angle annular dark-field (HAADF) image and corresponding EDS results of CoFe nanoparticles with coke formation.

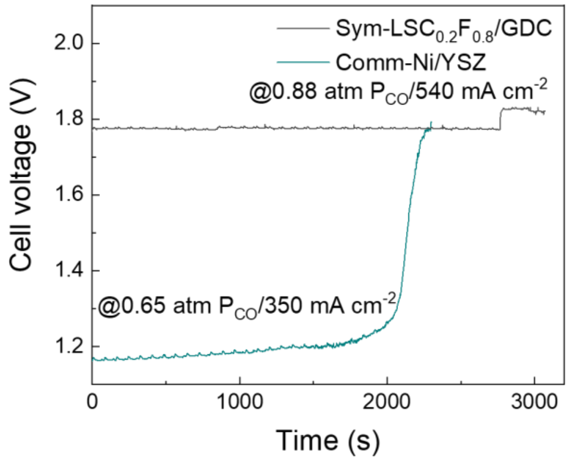


Figure S3. The voltage vs. time (V-t) profiles of the sym-$LSC_{0.2}F_{0.8}$/GDC and comm-Ni/YSZ cells at the threshold $P_{CO}$ for coke formation.

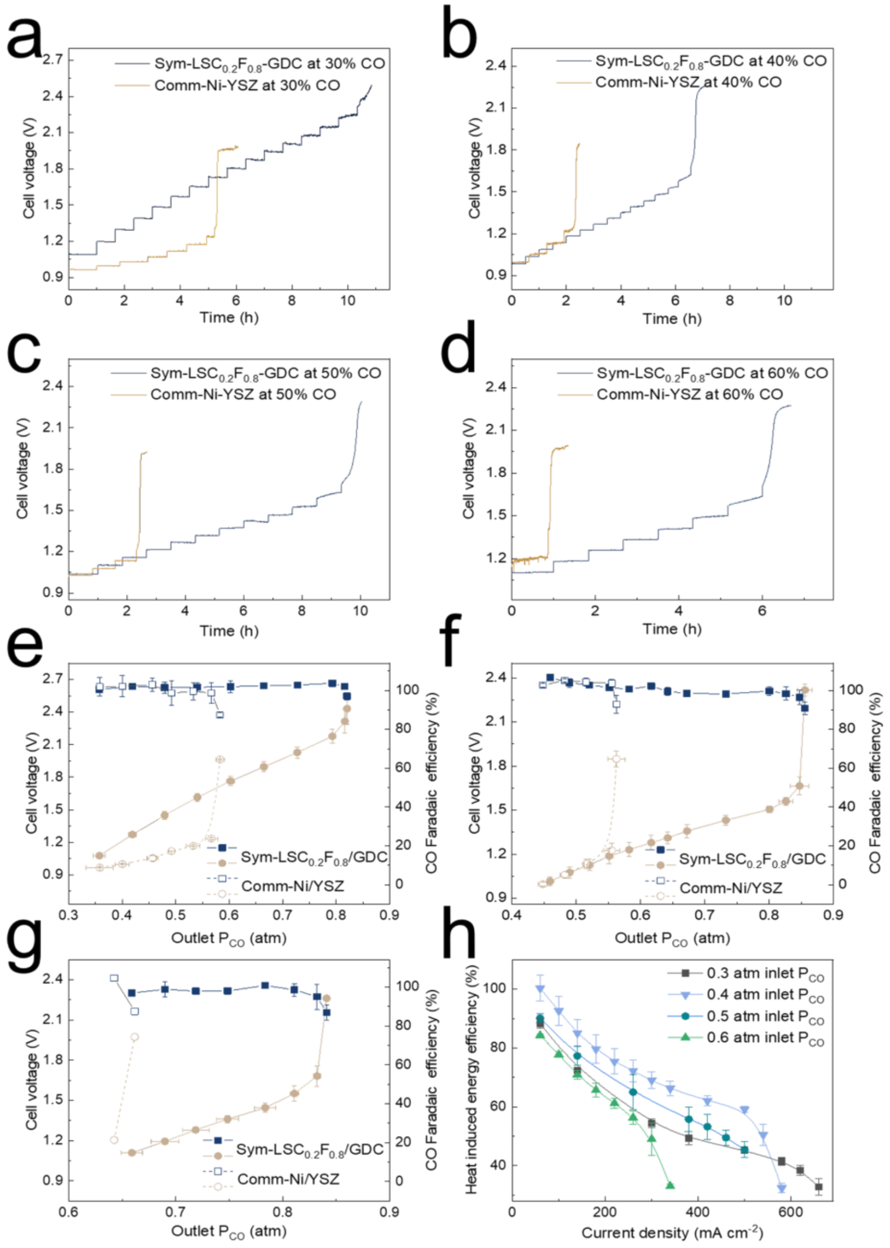


Figure S4. Detailed $CO_2RR$ performance on sym-$LSC_{0.2}F_{0.8}$/GDC and comm-Ni-YSZ cells at different inlet $P_{CO}$. (a-d) Voltage-time (V-t) profiles of sym-$LSC_{0.2}F_{0.8}$/GDC and comm-Ni-YSZ cells at different inlet $P_{CO}$. The initial current densities are 60 mA $cm^{-2}$, and each potential step corresponds to a 40 mA $cm^{-2}$ increase. (e-g) The cell voltages and CO FEs at different outlet $P_{CO}$. Increasing the current density increases the outlet $P_{CO}$ before coking. When $P_{CO}$ reaches the onset of coking, CO is converted into coke deposited at the electrode/electrolyte interface, causing the delamination of the cell and, thus, the jump of the cell voltage. Concurrently, a dynamic equilibrium consisting of $CO_2$-to-CO reduction and CO-to-coke conversion was established to flatten the $P_{CO}$ vs. cell voltage curve. Therefore, we found a near-constant $P_{CO}$ during coke formation. (h) The heat-included EEs.

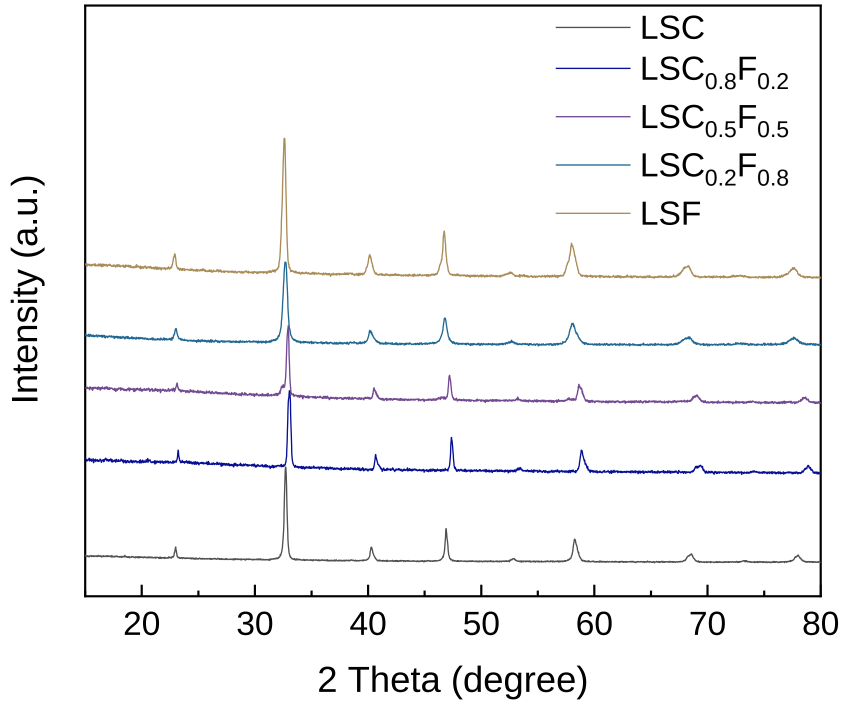


Figure S5. The XRD analysis of $LSC_{1-x}F_x$ (x = 0, 0.2, 0.5, 0.8, and 1).

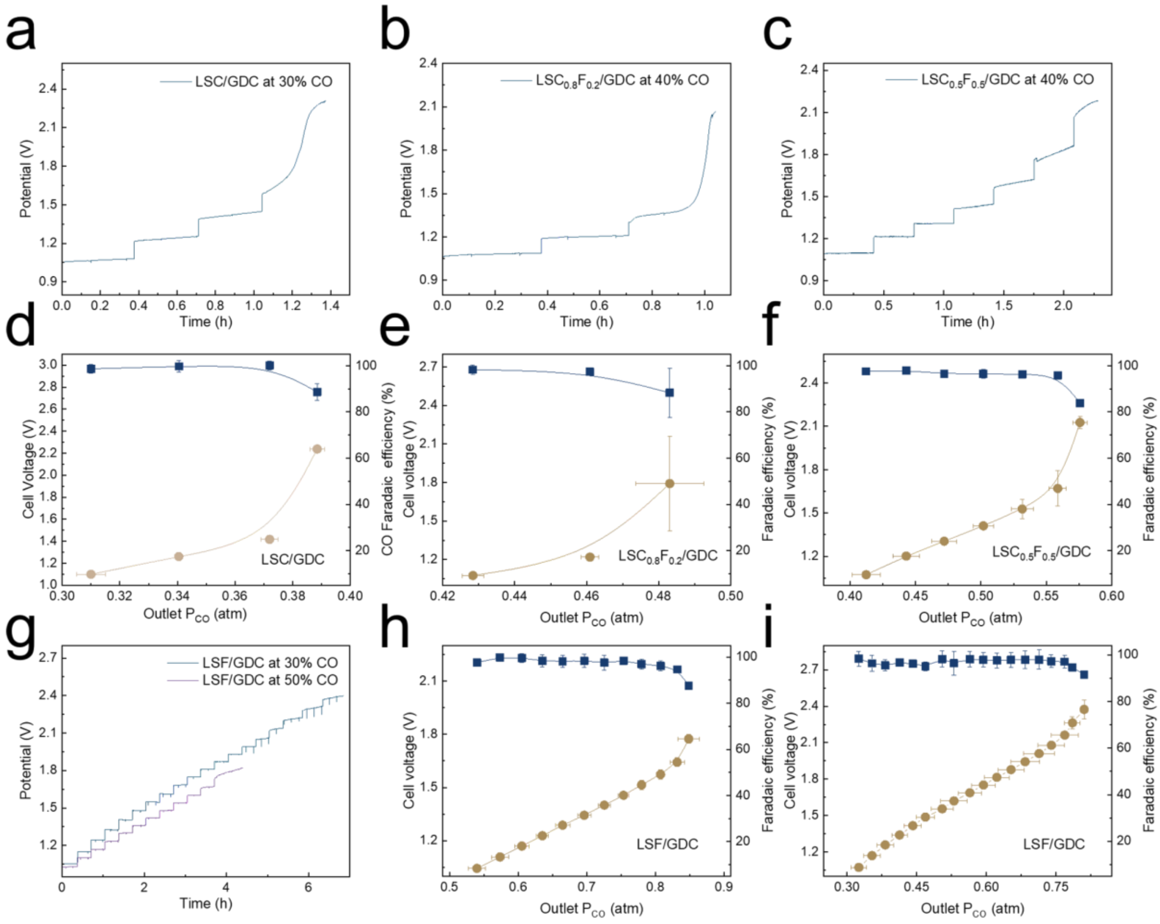


Figure S6. Detailed $CO_2$RR performance on $LSC_{1-x}F_x$/GDC cathodes at different inlet $P_{CO}$. (a-c) Voltage-time (V-t) profiles of LSC/GDC, $LSC_{0.8}F_{0.2}$/GDC, and $LSC_{0.5}F_{0.5}$/GDC, respectively. (d-f) The cell voltages and CO FEs of LSC/GDC, $LSC_{0.8}F_{0.2}$/GDC and $LSC_{0.5}F_{0.5}$/GDC at different outlet $P_{CO}$. (g-i) Voltage-time (V-t) profiles of LSF/GDC and the CO concentration at different voltages and outlet $P_{CO}$. The initial current densities are 60 mA $cm^{-2}$, and each potential step corresponds to a 40 mA $cm^{-2}$ increase.

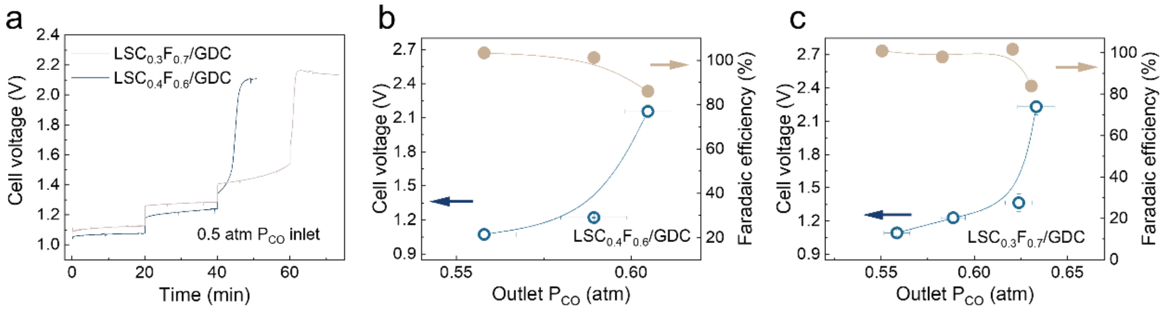


Figure S7. The $CO_2RR$ performance of $LSC_{0.3}F_{0.7}$/GDC and $LSC_{0.4}F_{0.6}$/GDC cells. (a) Voltage-time (V-t) profiles recorded at an initial current density of 60 mA $cm^{-2}$. Each subsequent potential step corresponds to an incremental increase of 60 mA $cm^{-2}$. (b) The cell voltages and CO FEs at different outlet $P_{CO}$. The inlet $P_{CO}$ was 0.5 atm inlet, and the active area of each cell was 5 $cm^2$.

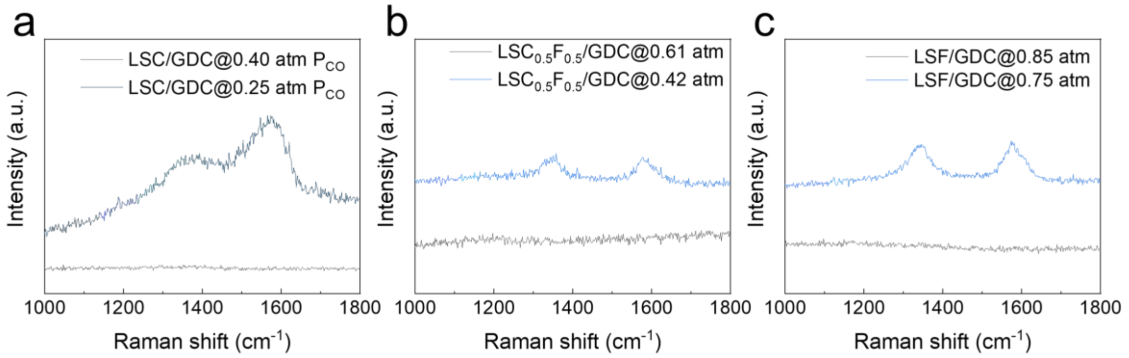


Figure S8. Raman spectra for (a) LSC/GDC, (b) $LSC_{0.5}F_{0.5}$/GDC, and (c) LSF/GDC electrodes. All samples were tested at 60 mA cm$^{-2}$. Two outlet $P_{CO}$ were used based on the results in Figure 1d to control whether coke formation occurs during the measurements.

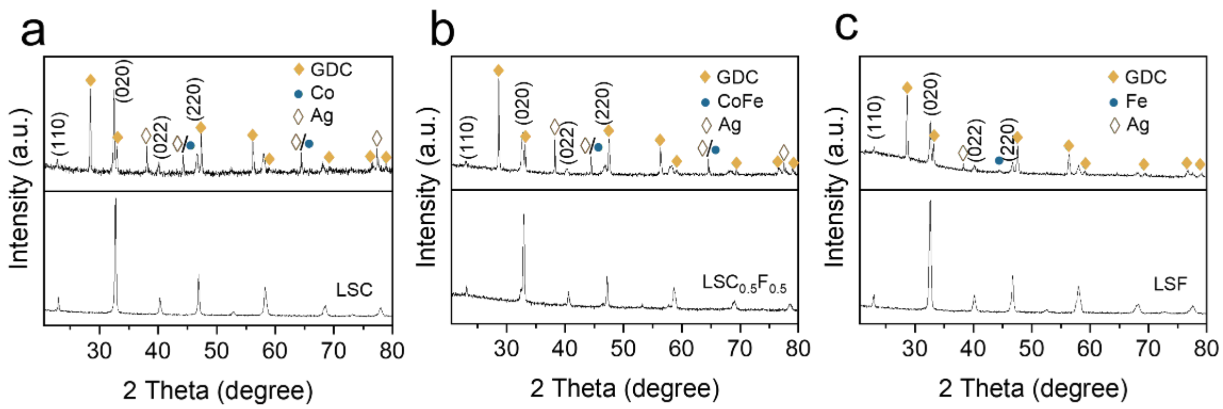


Figure S9. Structural evolution of LSC, $LSC_{0.5}F_{0.5}$, and LSF. (a-c) XRD analyses of the LSC/GDC, $LSC_{0.5}F_{0.5}$/GDC, and LSF/GDC cells before and after HT-$CO_2$RR. The applied current density was 60 mA $cm^2$, and the duration of each experiment was 20 minutes.

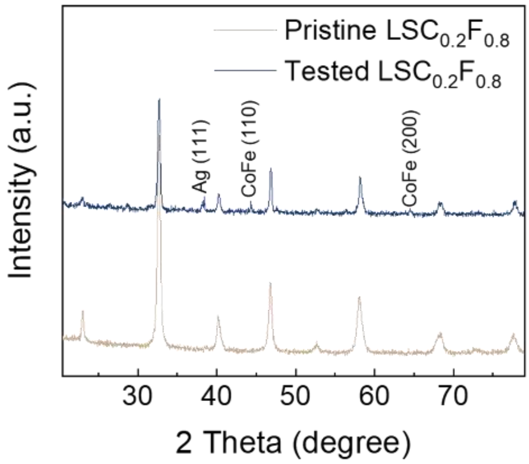


Figure S10. The XRD analysis of the sym-$LSC_{0.2}F_{0.8}$ cell using $La_{0.8}Sr_{0.2}Ga_{0.8}Mg_{0.2}O_{3-\delta}$ electrolyte before and after 80-min HT-$CO_2$RR.

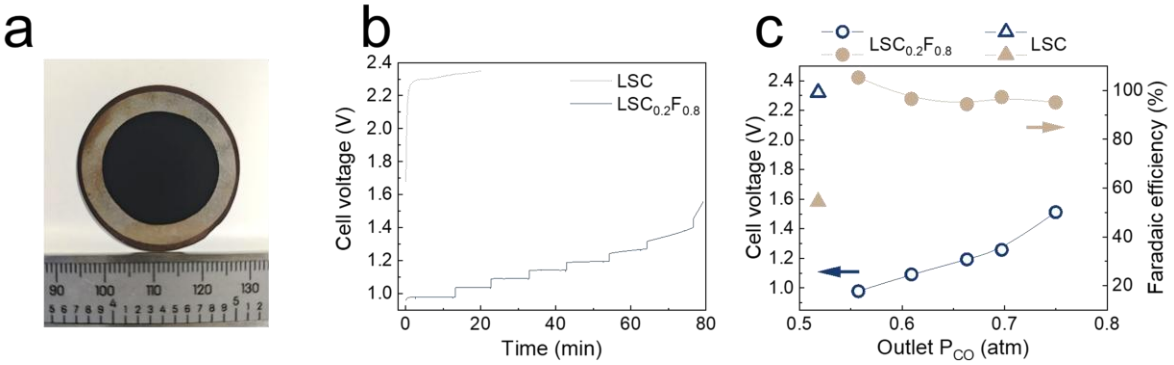


Figure S11. Cells using $La_{0.8}Sr_{0.2}Ga_{0.8}Mg_{0.2}O_{3-\delta}$ (LSGM) electrolyte. (a) The optical image of the $LSC_{1-x}F_x$|LSGM|$LSC_{0.2}F_{0.8}$ cell. The light-colored layer was formed by calcining the coated LSGM at 1200°C for 2 hours to reduce the contact resistance between the catalysts and the electrolyte. (b) Voltage-time (V-t) profiles of LSC and $LSC_{0.2}F_{0.8}$ cells, with an active area of 5 $cm^2$. (c) Cell voltages and CO Faradaic efficiencies (FEs) at different outlet $P_{CO}$. Increasing the current density leads to an increase in the outlet $P_{CO}$ before coking occurs. The LSGM electrolyte was fabricated by calcining at 1300°C for 5 hours.

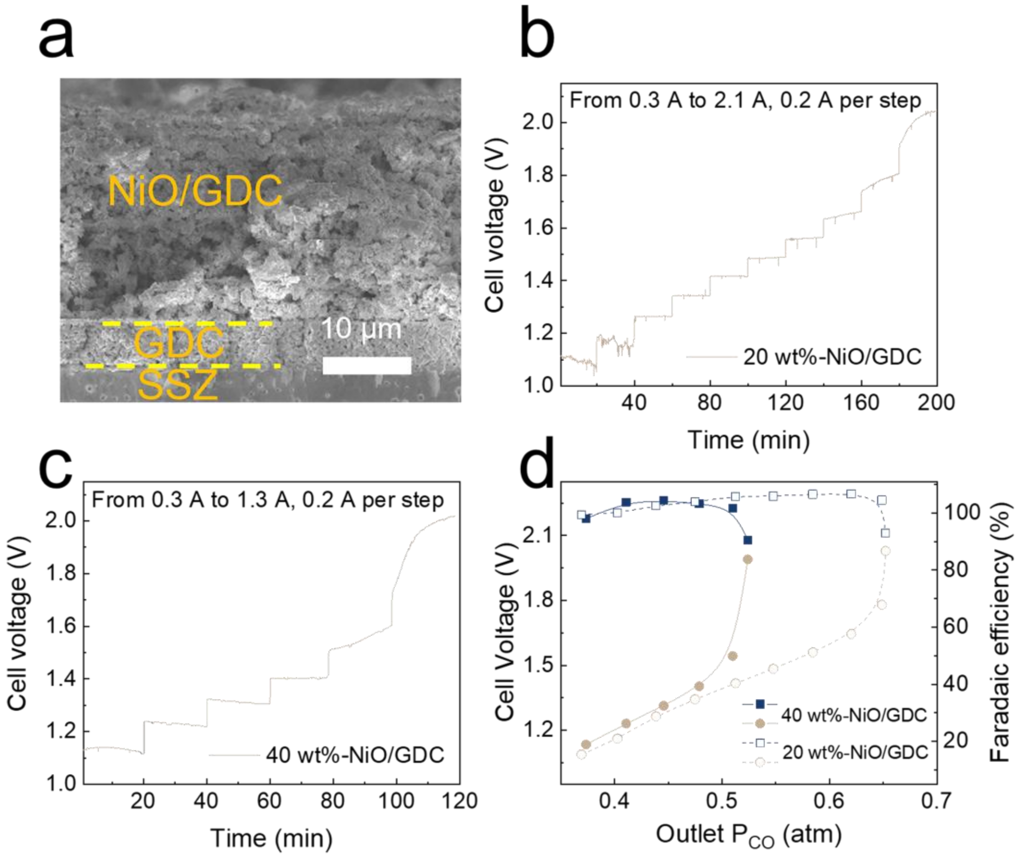


Figure S12. NiO/GDC Performance. (a) The cross-section SEM images of the NiO/GDC cell. (b) Voltage-time (V-t) profile of 20 wt%-NiO/GDC with an active area of 5 $cm^2$. (c) Voltage-time (V-t) profile of 40 wt%-NiO/GDC with an active area of 5 $cm^2$. (d) Cell voltages and CO faradaic efficiencies (FEs) at different outlet $P_{CO}$. Increasing the current density raises the outlet $P_{CO}$ before coking occurs.

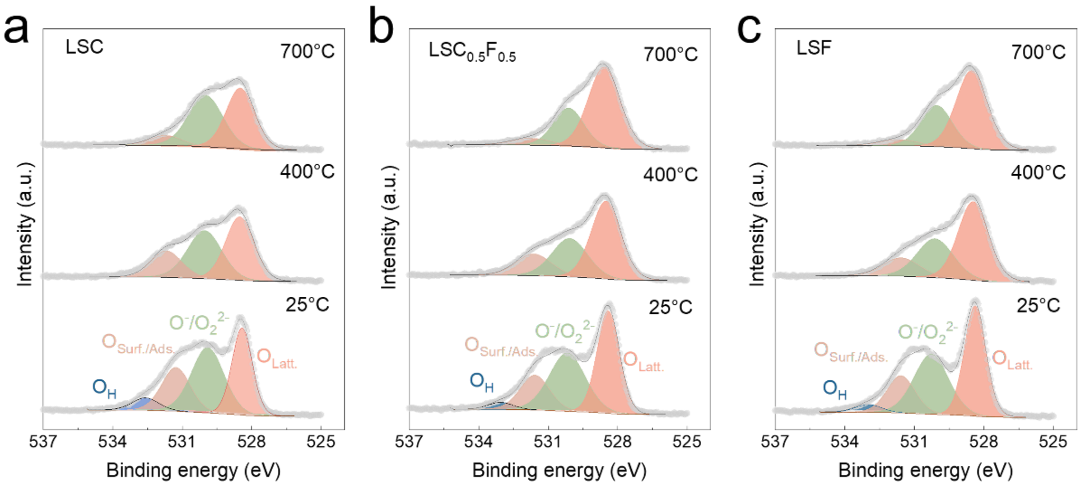


Figure S13. O 1s XPS spectra of as-prepared (a) LSC/GDC, (b) $LSC_{0.5}F_{0.5}$/GDC, and (c) LSF/GDC at 25°C, 400°C, and 700°C, respectively.

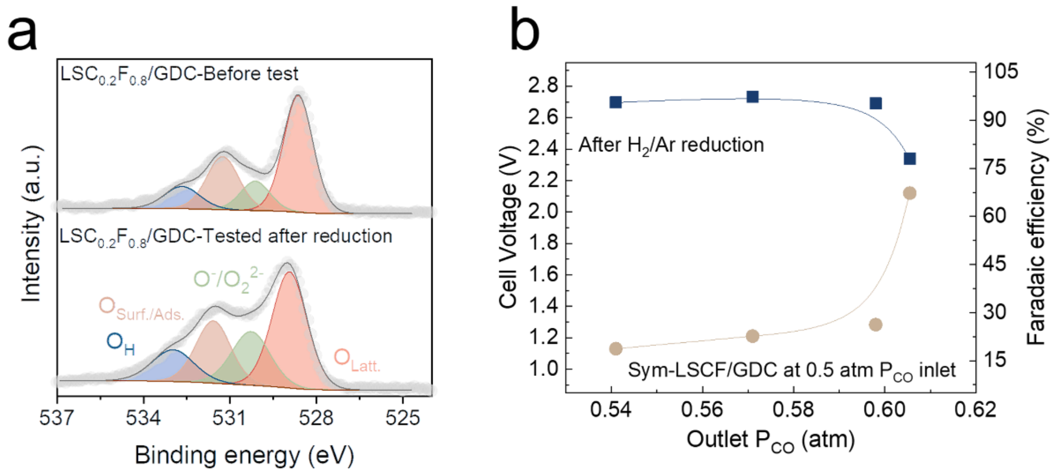


Figure S14. (a) O 1s XPS spectra of as-prepared $LSC_{0.2}F_{0.8}$/GDC and 10% $H_2$/Ar treated $LSC_{0.2}F_{0.8}$/GDC, respectively. (b) The cell voltages and CO FEs at different outlet $P_{CO}$.

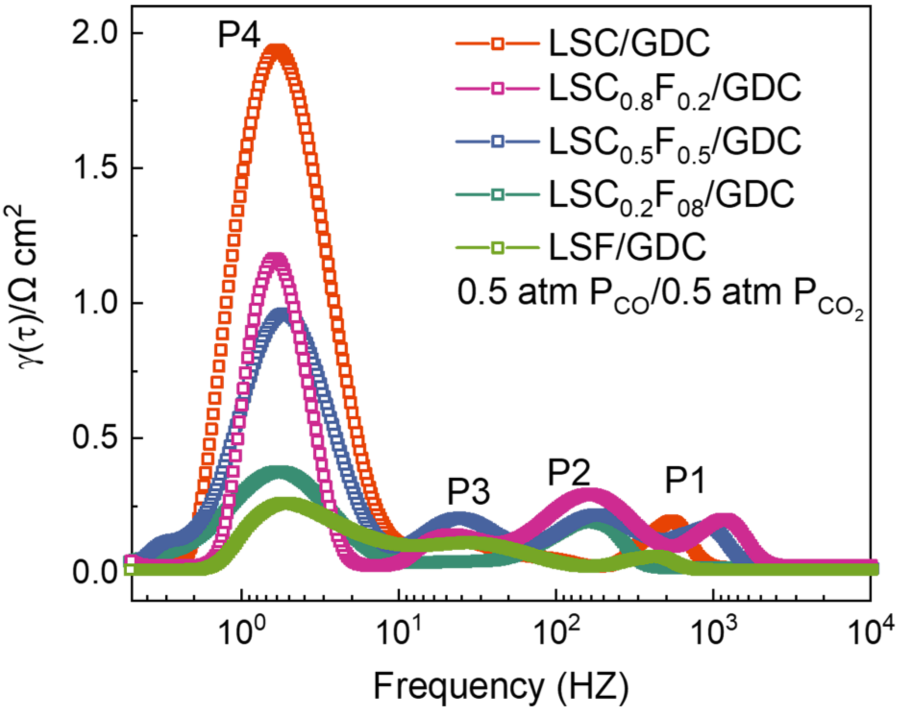


Figure S15. DRT plots for the $LSC_{1-x}F_x$/GDC electrode at 800°C and OCVunder mixed gas of $CO_2$/CO = 50/50.

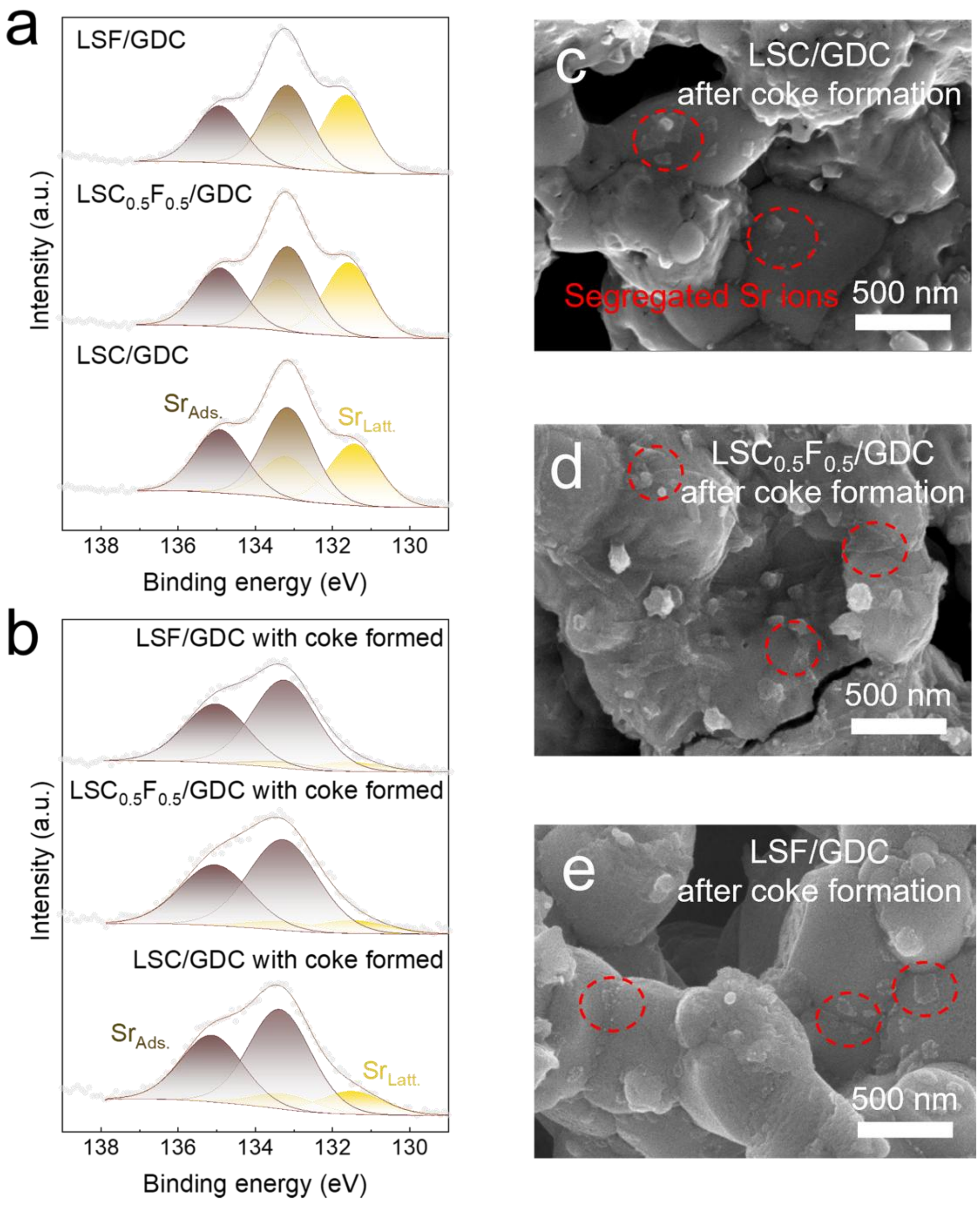


Figure S16. The impact of Sr segregation. (a) Sr 3d XPS spectra of as-prepared LSC/GDC, $LSC_{0.5}F_{0.5}$/GDC, and LSF/GDC cells. (b) Sr 3d XPS spectra of LSC/GDC, $LSC_{0.5}F_{0.5}$/GDC, and LSF/GDC cells with coke formation. (c-e) SEM images of LSC/GDC, $LSC_{0.5}F_{0.5}$/GDC, and LSF/GDC with coke formation. Sr ions segregated to the surface are circled in red.

Table S1. Proportion of Sr species before and after the test.

| Samples | Conditions | $Sr_{latt.}$ | $Sr_{ads.}$ |
|---|---|---|---|
| LSC/GDC | Pristine | 0.40 | 0.60 |
| | Coke formed | 0.19 | 0.81 |
| $LSC_{0.5}F_{0.5}$/GDC | Pristine | 0.46 | 0.54 |
| | Coke formed | 0.12 | 0.88 |
| LSF/GDC | Pristine | 0.48 | 0.52 |
| | Coke formed | 0.10 | 0.90 |

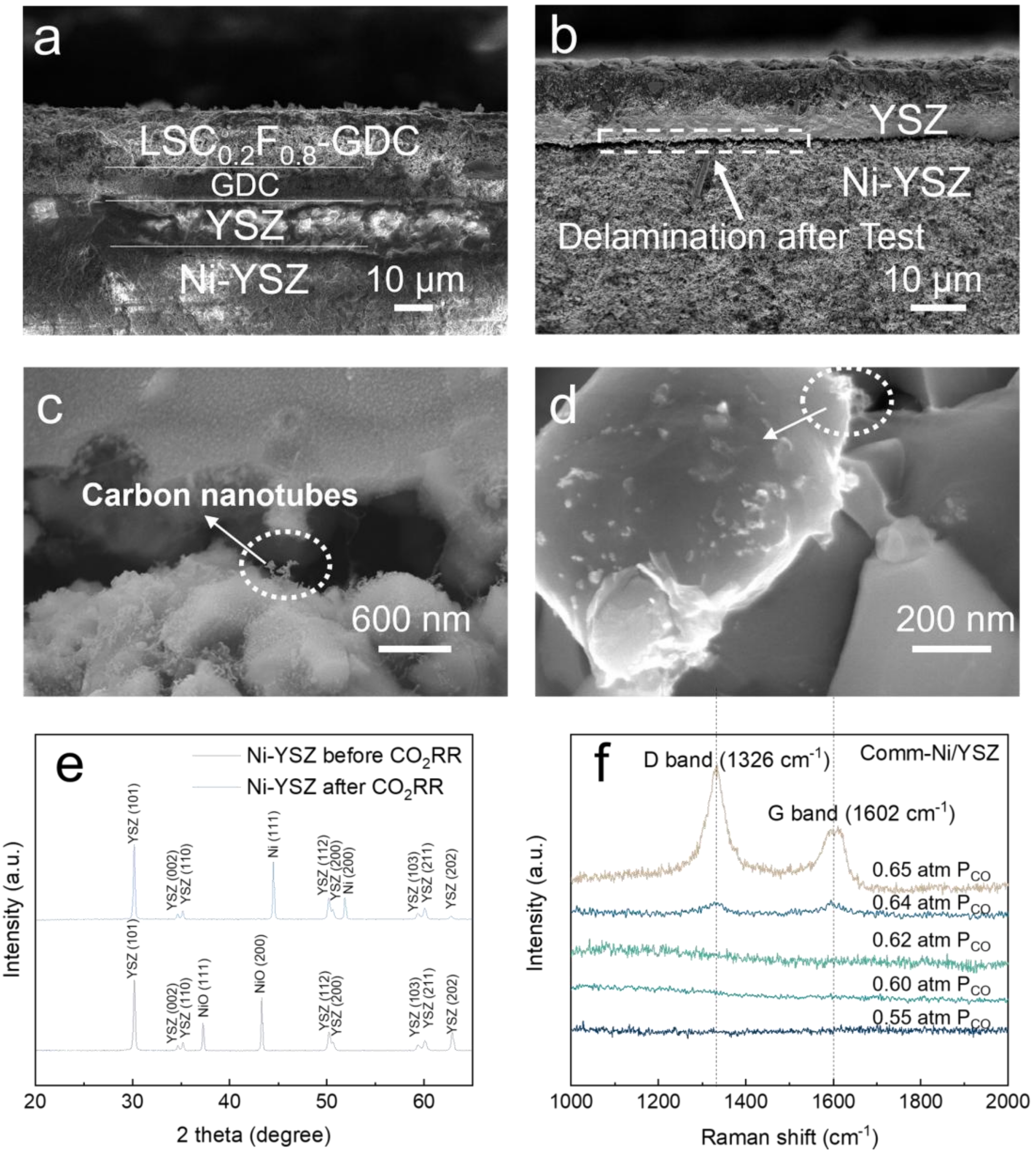


Figure S17. Ex-situ studies on the coke tolerance of the comm-Ni/YSZ cell. (a) The cross-section SEM image of the comm-Ni/YSZ cell before testing. (b) The cross-section SEM image of the comm-Ni/YSZ cell with coke-formation-caused delamination after testing. (c and d) SEM images of coke formation at the electrolyte/electrode interface and the surface of Ni/YSZ. (e) The XRD patterns of the comm-Ni/YSZ cell before and after HT-$CO_2$RR. (f) Raman analyses of the comm-Ni/YSZ cell after testing at different outlet $P_{CO}$.

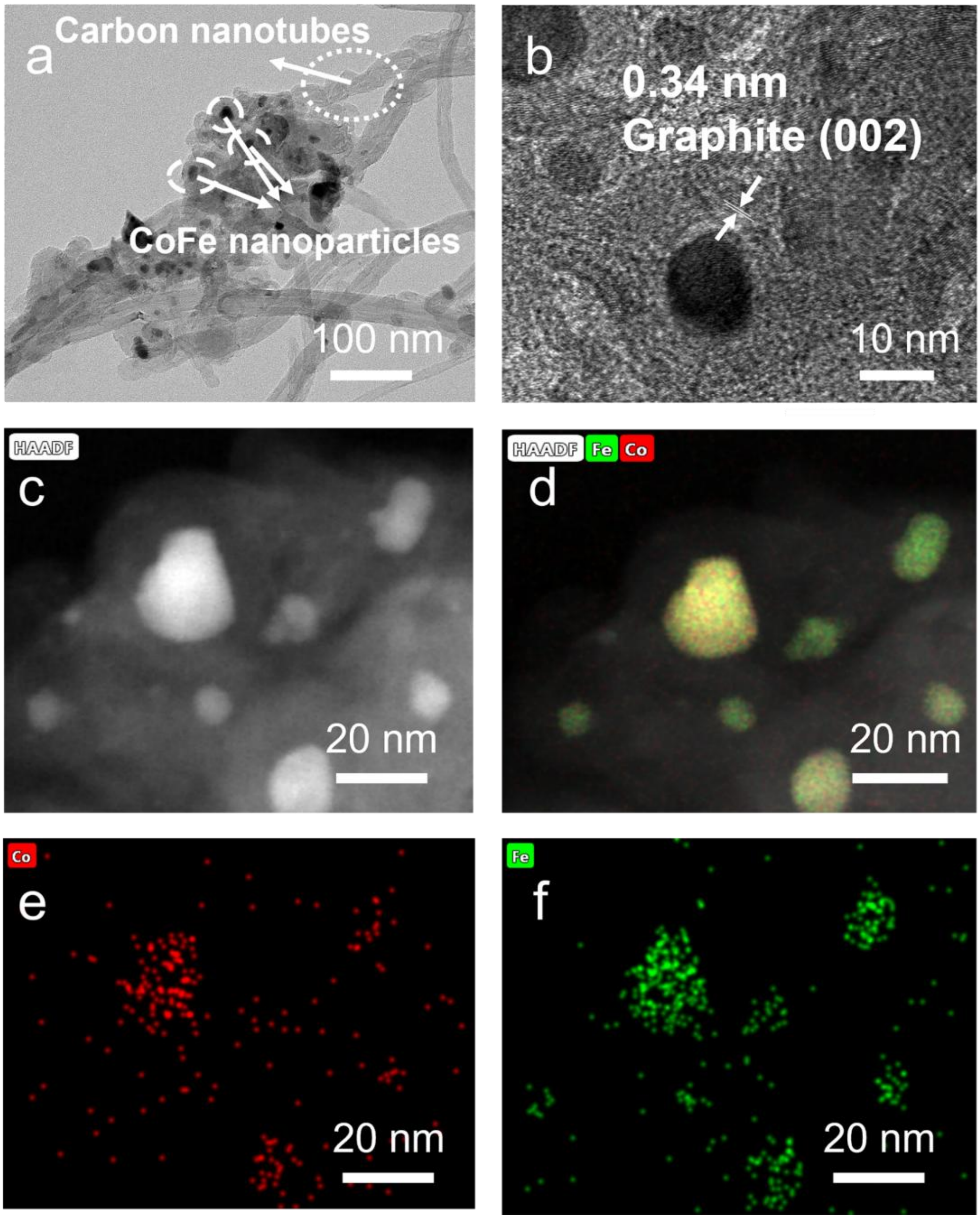


Figure S18. The TEM characterization of carbon encapsulated CoFe nanoparticles after coke formation (~1.8 V and ~0.87 atm $P_{CO}$). (a) TEM images of carbon nanotube encapsulated CoFe particles. (b) HRTEM images of the CoFe nanoparticles. (c-f) The high-angle annular dark-field (HAADF) image and corresponding EDS results of CoFe nanoparticles. The inlet $P_{CO}$ was fixed at ~0.5 atm with a total $CO/CO_2$ flow rate of ~50 sccm.

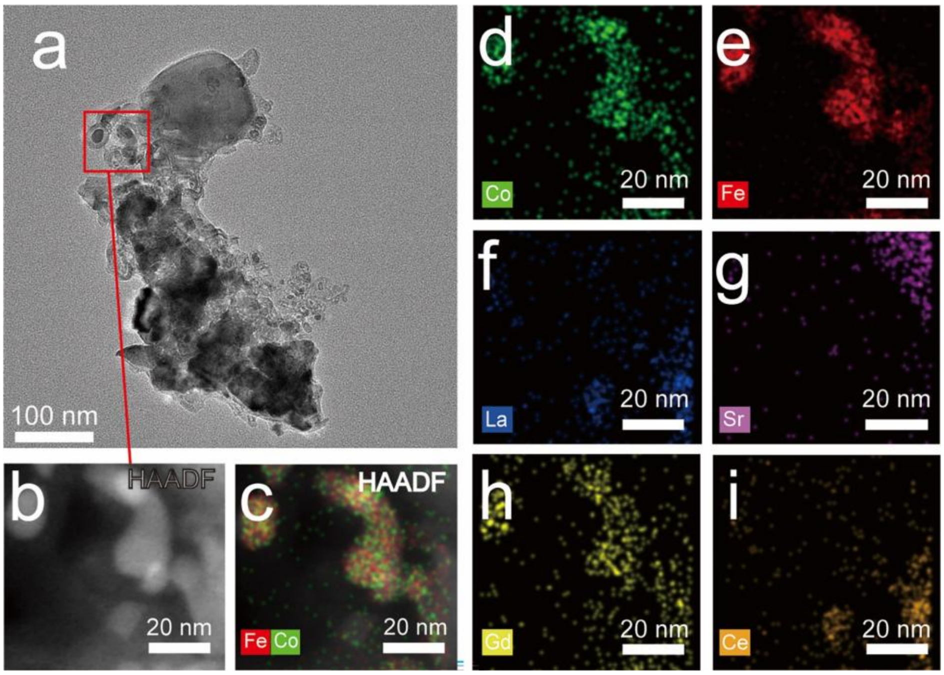


Figure S19. The TEM characterization of $LSC_{0.2}F_{0.8}$-GDC particles after testing at a 0.85 atm $P_{CO}$ and ~2.3 V. (a) The TEM, HAADF images, and elemental maps of $LSC_{0.2}F_{0.8}$/GDC particles with coke formation. (b-i) The corresponding HAADF image and elemental maps of the same $LSC_{0.2}F_{0.8}$/GDC particles in the red box. The inlet $P_{CO}$ was fixed at ~0.3 atm with a total $CO/CO_2$ flow rate of ~50 sccm.

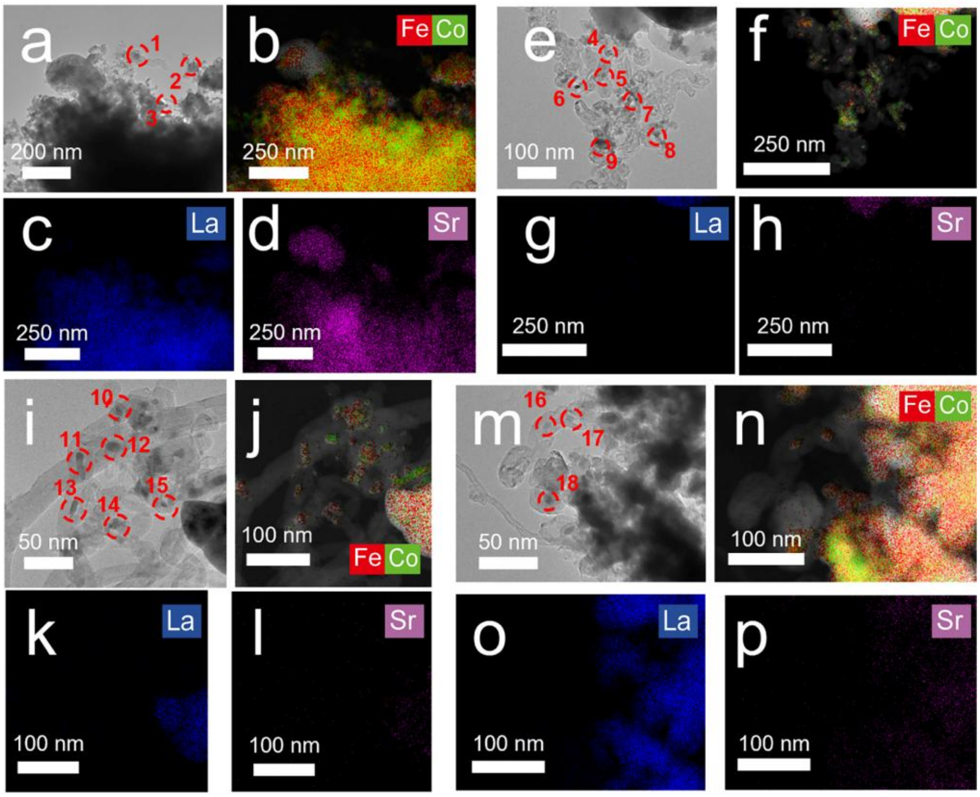


Figure S20. The TEM characterization of carbon encapsulated CoFe nanoparticles after coke formation (~1.9 V, ~0.86 atm $P_{CO}$). 18 random spots were studied. Carbon-encapsulated metal nanoparticles were highlighted using red circles. The inlet $P_{CO}$ was fixed at ~0.5 atm with a total $CO/CO_2$ flow rate of ~50 sccm.

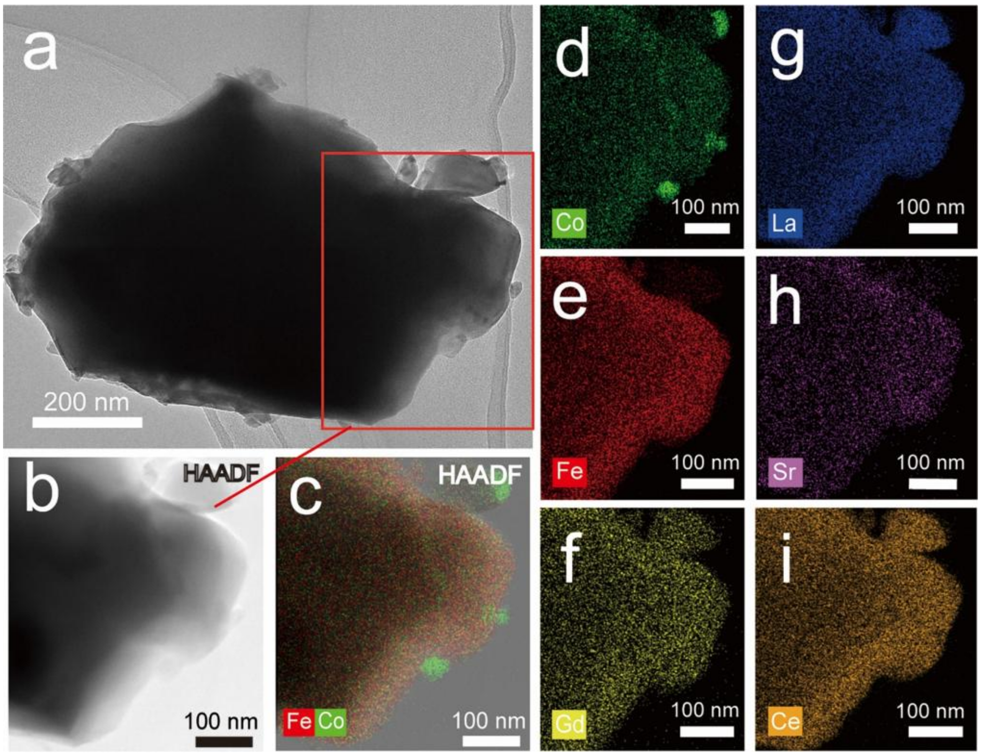

Figure S21. The TEM characterization of $LSC_{0.2}F_{0.8}$-GDC particles after testing at a 0.80 atm $P_{CO}$ and ~1.5 V. (a) The TEM, HAADF images, and elemental maps of $LSC_{0.2}F_{0.8}$/GDC particles without coke formation. (b-i) The corresponding HAADF image and elemental maps of the same $LSC_{0.2}F_{0.8}$/GDC particles in the red box. The inlet $P_{CO}$ was fixed at ~0.5 atm with a total $CO/CO_2$ flow rate of ~50 sccm.

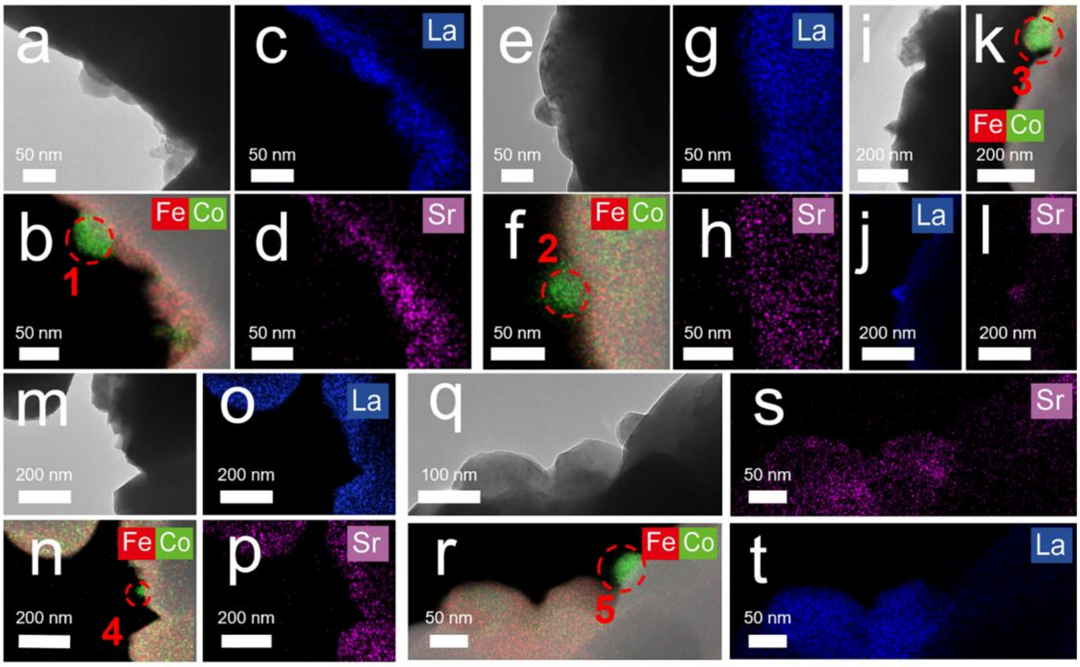


Figure S22. The TEM characterization of $LSC_{0.2}F_{0.8}$-GDC particles after testing at 0.78 atm $P_{CO}$ and ~1.8 V. 5 random spots were studied. Exsolved metal nanoparticles after $CO_2$RR were highlighted using red circles. The inlet $P_{CO}$ was fixed at ~0.5 atm with a total $CO/CO_2$ flow rate of ~50 sccm.

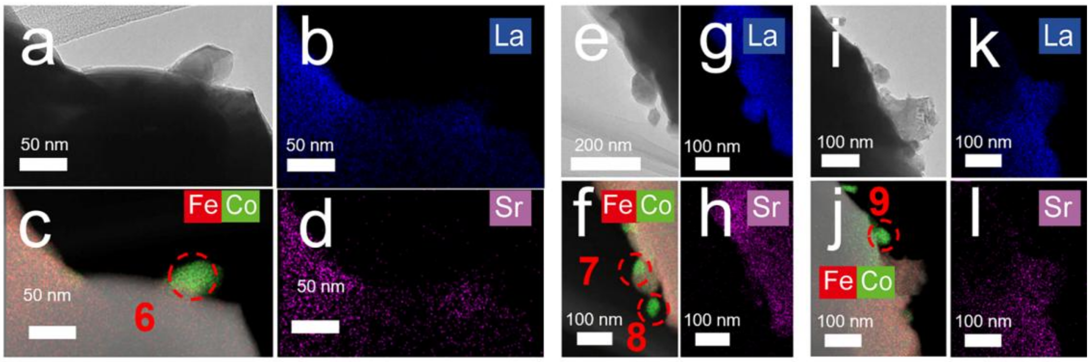


Figure S23. Additional TEM characterization of $LSC_{0.2}F_{0.8}$-GDC particles after testing at 0.78 atm $P_{CO}$ and ~1.8 V. 4 more random spots were studied. Exsolved metal nanoparticles after $CO_2RR$ were highlighted using red circles. The inlet $P_{CO}$ was fixed at ~0.5 atm with a total $CO/CO_2$ flow rate of ~50 sccm.

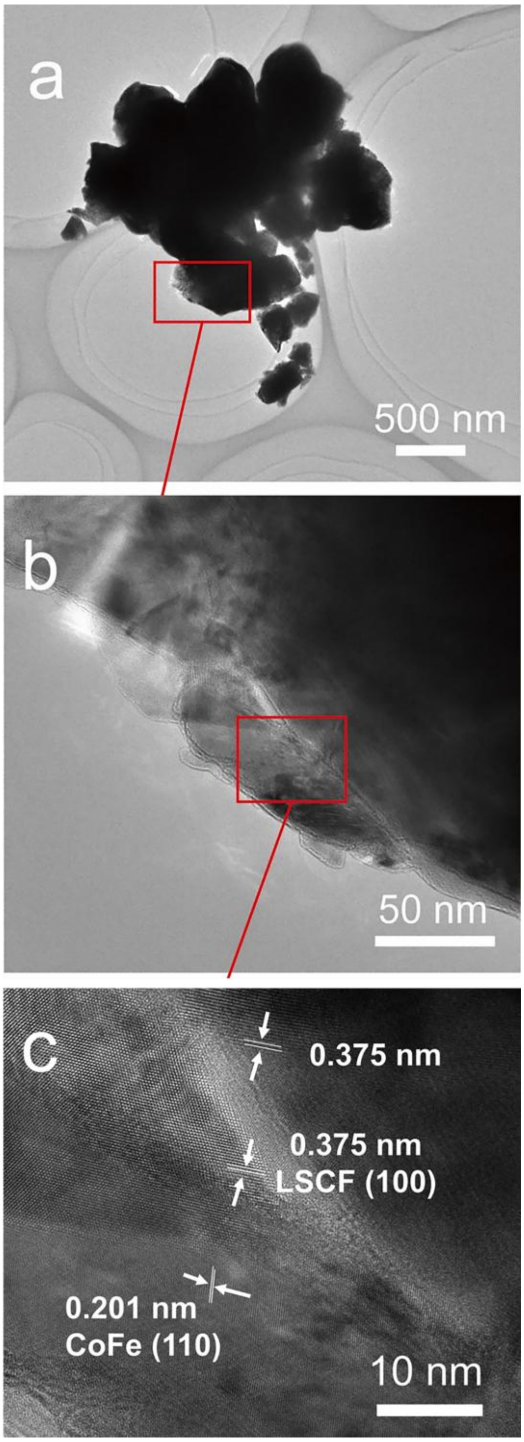

Figure S24. TEM images of $LSC_{0.2}F_{0.8}$-GDC particles after testing at a 0.72 atm $P_{CO}$ and ~2 V. (a and b) TEM images of $LSC_{0.2}F_{0.8}$/GDC particles after HT-$CO_2$RR under different magnifications. (c) The HRTEM images of exsolved CoFe particles after HT-$CO_2$RR.

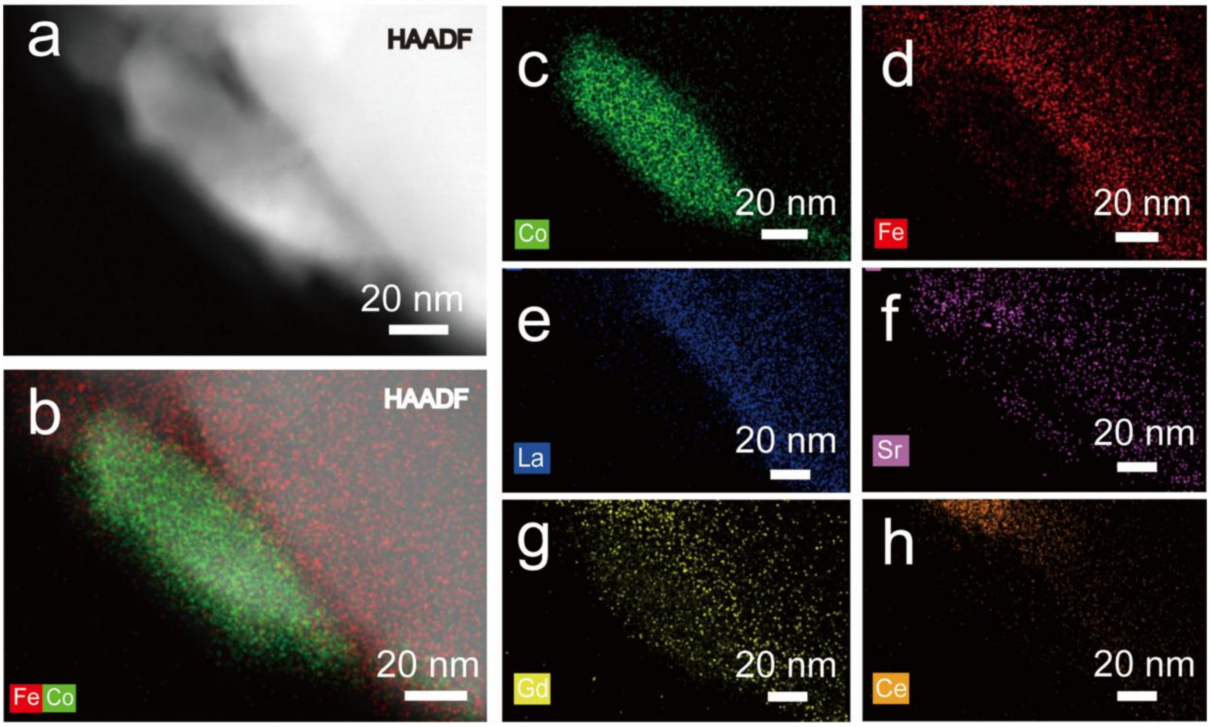


Figure S25. The element distribution in $LSC_{0.2}F_{0.8}$-GDC particles after testing at a 0.72 atm $P_{CO}$ and ~2 V. (a) The HAADF images, and (b-h) the elemental maps of a representative exsolved CoFe nanoparticle. The inlet $P_{CO}$ was fixed at ~0.3 atm with a total $CO/CO_2$ flow rate of ~50 sccm.

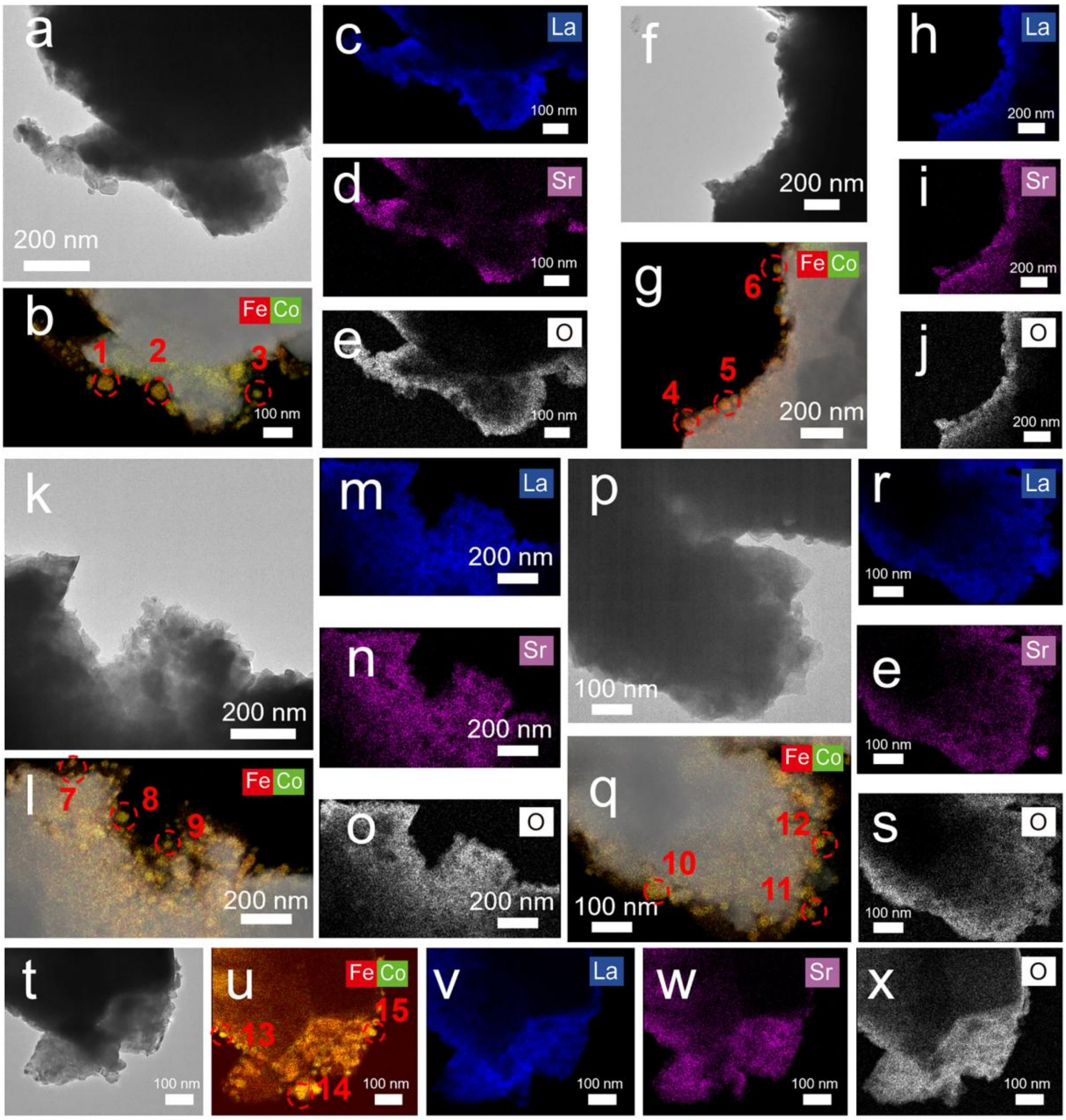


Figure S26. The characterization of $LSC_{0.2}F_{0.8}$-GDC particle treated in 10%$H_2$/Ar atmosphere at 800$^o$C. 15 random spots were studied. Exsolved metal nanoparticles after $CO_2$RR were highlighted using red circles.

Table S2. The Fe and Co atomic fractions in Figures S2, S18, S19, S21, and S25.

| Sample | Element | Family | Atomic Fraction (%) | Mass Fraction (%) |
|---|---|---|---|---|
| Figure S2c, d | Fe | K | 10.66 | 3.4 |
| | Co | K | 89.34 | 21.27 |
| Figure S2e, f | Fe | K | 92.57 | 15.72 |
| | Co | K | 7.43 | 1.77 |
| Figure S18 (CoFe) | Fe | K | 74.2 | 48.4 |
| | Co | K | 25.8 | 17.7 |
| Figure S19 ($LSC_{0.2}F_{0.8}$) | Fe | K | 28.4 | 39.7 |
| | Co | K | 0.5 | 0.7 |
| Figure S19 (CoFe) | Fe | K | 87.5 | 87.0 |
| | Co | K | 12.5 | 13.1 |
| Figure S21 ($LSC_{0.2}F_{0.8}$) | Fe | K | 20.5 | 24.3 |
| | Co | K | 0.7 | 0.9 |
| Figure S21 (CoFe) | Fe | K | 5.0 | 3.1 |
| | Co | K | 95.0 | 61.9 |
| Figure S25 (CoFe) | Fe | K | 5.3 | 3.6 |
| | Co | K | 94.7 | 67.7 |
| Figure S25 ($LSC_{0.2}F_{0.8}$) | Fe | K | 24.9 | 24.5 |
| | Co | K | 1.6 | 1.7 |

Table S3. The Fe and Co atomic fractions in Figure S20.

| | | Element | Family | Atomic Fraction (%) | Mass Fraction (wt%) | wt% Sigma |
|---|---|---|---|---|---|---|
| S20 | 1 | Fe | K | 53.60 | 52.25 | 1.43 |
| | | Co | K | 46.40 | 47.75 | 1.32 |
| | 2 | Fe | K | 72.15 | 68.51 | 0.62 |
| | | Co | K | 27.85 | 31.49 | 0.51 |
| | 3 | Fe | K | 67.41 | 67.41 | 2.69 |
| | | Co | K | 32.59 | 32.59 | 1.34 |
| | 4 | Fe | K | 47.65 | 46.30 | 1.41 |
| | | Co | K | 52.35 | 53.70 | 1.46 |
| | 5 | Fe | K | 74.79 | 73.72 | 2.04 |
| | | Co | K | 25.21 | 26.28 | 1.51 |
| | 6 | Fe | K | 48.64 | 47.33 | 2.95 |
| | | Co | K | 51.36 | 52.67 | 3.26 |
| | 7 | Fe | K | 53.70 | 52.34 | 1.58 |
| | | Co | K | 46.30 | 47.66 | 1.46 |
| | 8 | Fe | K | 48.06 | 46.82 | 0.76 |
| | | Co | K | 51.94 | 53.18 | 0.80 |
| | 9 | Fe | K | 56.59 | 55.33 | 0.88 |
| | | Co | K | 43.41 | 44.67 | 0.77 |
| | 10 | Fe | K | 39.00 | 37.73 | 2.97 |
| | | Co | K | 61.00 | 62.27 | 4.72 |
| | 11 | Fe | K | 71.36 | 70.30 | 1.44 |
| | | Co | K | 28.64 | 29.70 | 1.21 |
| | 12 | Fe | K | 23.33 | 22.42 | 0.72 |
| | | Co | K | 76.67 | 77.58 | 2.07 |

| | | | | | |
|---|---|---|---|---|---|
| 13 | Fe | K | 69.25 | 68.07 | 5.03 |
| | Co | K | 30.75 | 31.93 | 2.50 |
| 14 | Fe | K | 88.44 | 88.05 | 1.30 |
| | Co | K | 11.56 | 11.95 | 0.72 |
| 15 | Fe | K | 70.30 | 69.12 | 6.64 |
| | Co | K | 29.70 | 30.88 | 3.12 |
| 16 | Fe | K | 83.56 | 82.77 | 0.86 |
| | Co | K | 16.44 | 17.23 | 0.67 |
| 17 | Fe | K | 82.69 | 81.93 | 0.85 |
| | Co | K | 17.31 | 18.07 | 0.70 |
| 18 | Fe | K | 78.67 | 77.80 | 0.53 |
| | Co | K | 21.33 | 22.20 | 0.47 |

Table S4. The Fe and Co atomic fractions in Supplementary Figures S22 and 23.

| | | Element | Family | Atomic Fraction (%) | Atomic Error (%) |
|---|---|---|---|---|---|
| S22-23 | 1 | Fe | K | 7.38 | 0.48 |
| | | Co | K | 92.62 | 5.80 |
| | 2 | Fe | K | 6.17 | 0.62 |
| | | Co | K | 93.83 | 7.52 |
| | 3 | Fe | K | 7.68 | 0.56 |
| | | Co | K | 92.32 | 6.72 |
| | 4 | Fe | K | 11.90 | 0.81 |
| | | Co | K | 88.10 | 5.55 |
| | 5 | Fe | K | 15.27 | 0.84 |
| | | Co | K | 84.73 | 4.21 |
| | 6 | Fe | K | 3.85 | 0.31 |
| | | Co | K | 96.15 | 6.60 |
| | 7 | Fe | K | 5.61 | 0.36 |
| | | Co | K | 94.39 | 5.44 |
| | 8 | Fe | K | 6.78 | 0.36 |
| | | Co | K | 93.22 | 3.73 |
| | 9 | Fe | K | 8.98 | 0.26 |
| | | Co | K | 91.02 | 1.68 |

Table S5. The Fe and Co atomic fractions in Figure S26.

| | | Element | Family | Atomic Fraction (%) | Atomic Error (%) |
|---|---|---|---|---|---|
| S26 | 1 | Fe | K | 73.12 | 6.58 |
| | | Co | K | 26.88 | 2.43 |
| | 2 | Fe | K | 79.89 | 9.00 |
| | | Co | K | 20.11 | 2.27 |
| | 3 | Fe | K | 76.12 | 8.41 |
| | | Co | K | 23.88 | 2.64 |
| | 4 | Fe | K | 72.22 | 6.38 |
| | | Co | K | 27.78 | 2.47 |
| | 5 | Fe | K | 73.11 | 5.93 |
| | | Co | K | 26.89 | 2.19 |
| | 6 | Fe | K | 71.84 | 5.81 |
| | | Co | K | 28.16 | 2.29 |
| | 7 | Fe | K | 72.33 | 9.18 |
| | | Co | K | 27.67 | 3.53 |
| | 8 | Fe | K | 78.31 | 6.11 |
| | | Co | K | 21.69 | 1.74 |
| | 9 | Fe | K | 76.00 | 5.26 |
| | | Co | K | 24.00 | 1.68 |
| | 10 | Fe | K | 76.06 | 7.24 |
| | | Co | K | 23.94 | 2.29 |
| | 11 | Fe | K | 78.82 | 6.82 |
| | | Co | K | 21.18 | 1.89 |

| | | | | | |
|---|---|---|---|---|---|
| | 12 | Fe | K | 76.61 | 6.79 |
| | | Co | K | 23.39 | 2.08 |
| | 13 | Fe | K | 75.67 | 3.74 |
| | | Co | K | 25.33 | 1.28 |
| | 14 | Fe | K | 72.64 | 6.75 |
| | | Co | K | 27.36 | 2.55 |
| | 15 | Fe | K | 74.74 | 7.23 |
| | | Co | K | 25.26 | 2.46 |

Table S6. The ratio of Fe to Co in samples tested under different conditions. Data derived from TEM results are presented in Tables 2-5 of the Supporting Information.

| Condition | | The ratio of Fe to Co (error bar) |
|---|---|---|
| With coke formation | 1.8 V | 2.87 : 1 |
| | 1.9 V | 4.41 (±3.25) : 1 |
| | 2.3 V | 7 : 1 |
| Without coke formation | 1.5 V | 0.05: 1 |
| | 1.8 V | 0.11 (±0.07) : 1 |
| | 2 V | 0.06: 1 |
| 10% $H_2$/Ar treated | - | 3.26 (±0.71) : 1 |

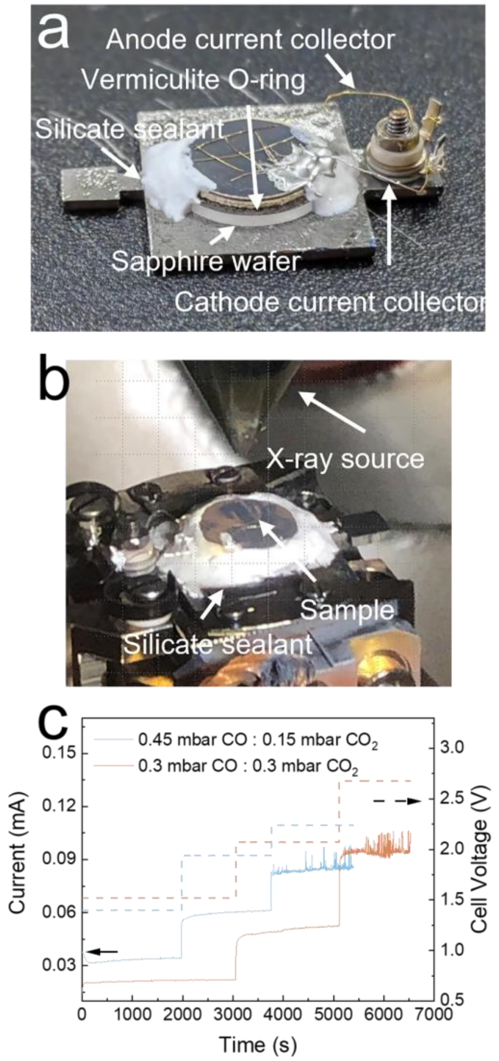


Figure S27. The NAP-XPS test. (a) The image of the model cell after in-situ NAP-XPS measurements. (b) The image of the model cell during the NAP-XPS test. (c) The current-time and voltage-time profiles under different $P_{CO}$:$P_{CO2}$ ratios.

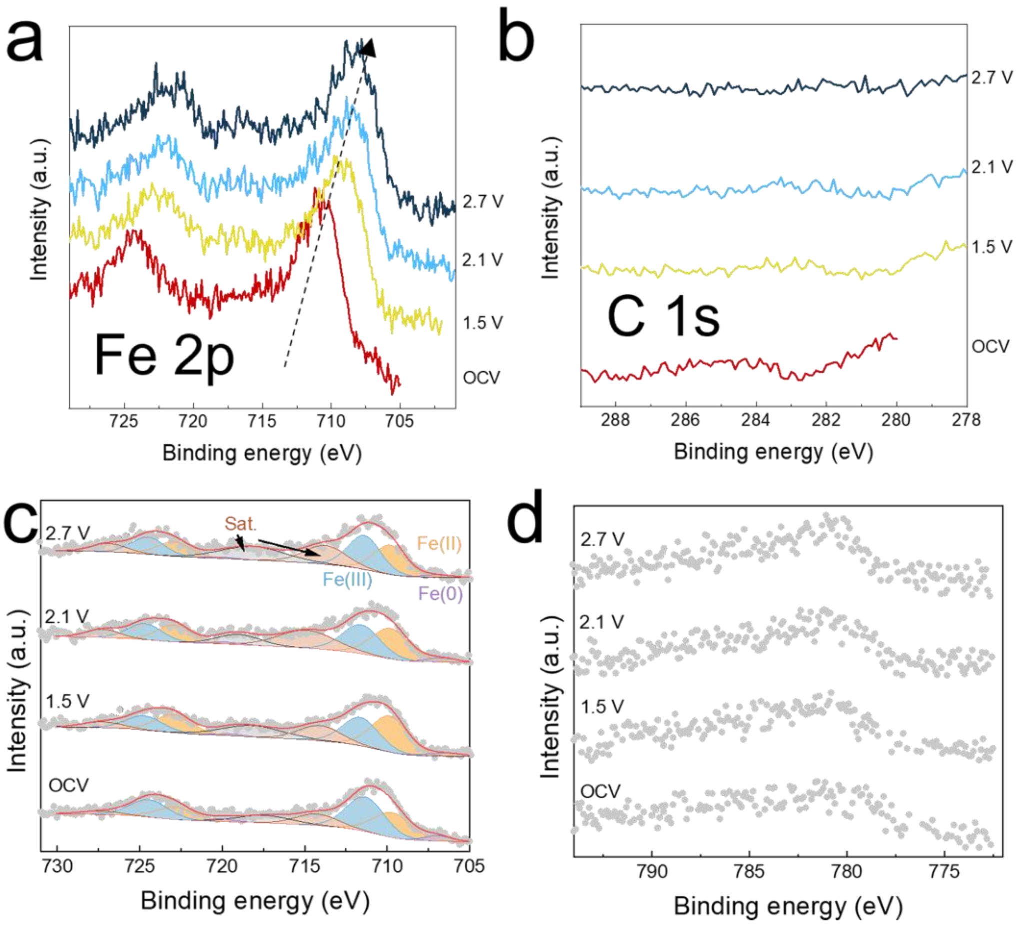


Figure S28. The NAP-XPS results at 0.3 mbar $P_{CO}$ and 0.3 mbar $P_{CO2}$. (a and b) The raw Fe 2p and C 1s NAP-XPS spectra at 0.3 mbar $P_{CO}$ and 0.3 mbar $P_{CO2}$. (c and d) The deconvoluted Fe 2p and raw Co 2p XPS spectra at 0.3 mbar $P_{CO}$ and 0.3 mbar $P_{CO2}$.

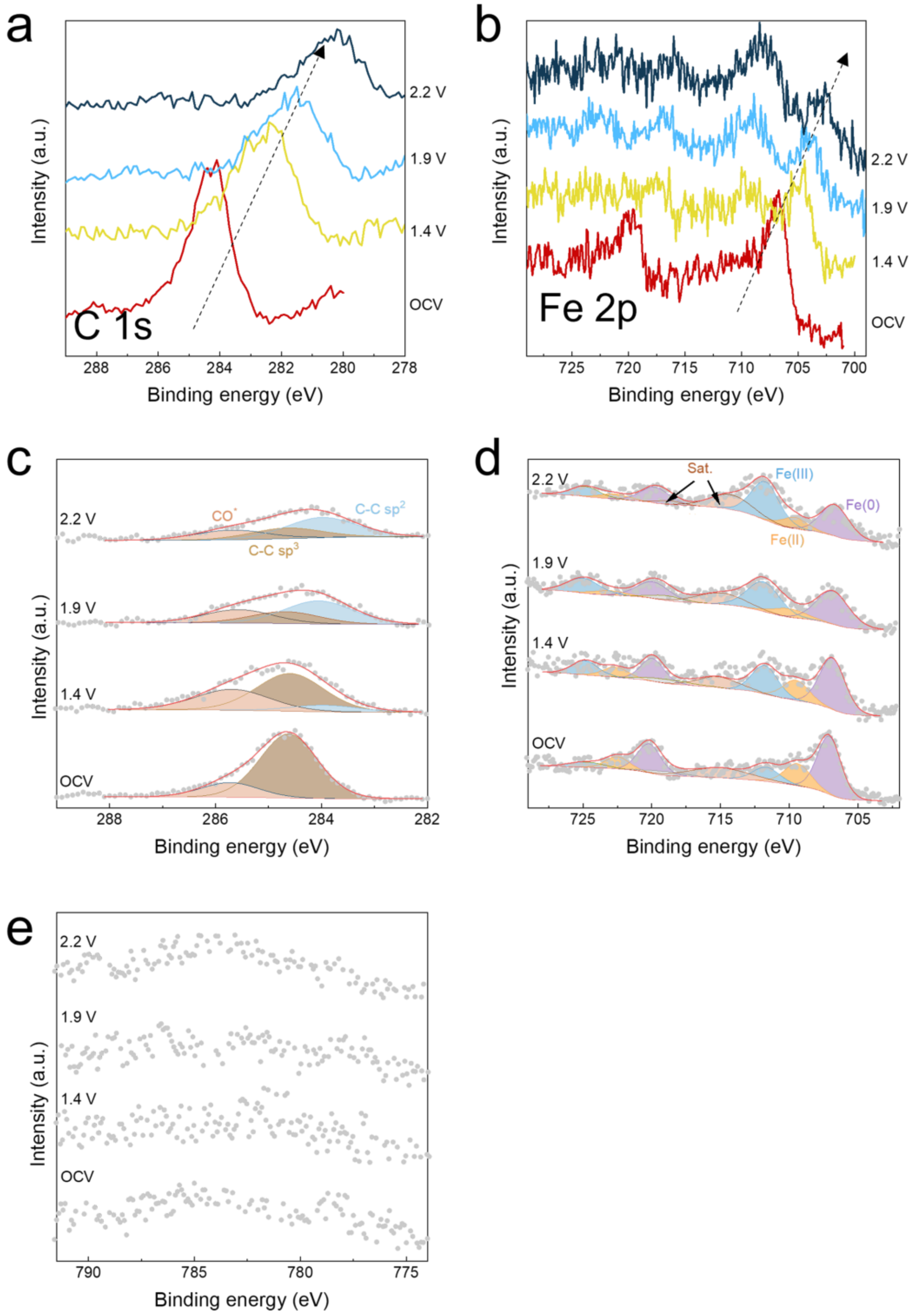


Figure S29. The NAP-XPS results at 0.45 mbar $P_{CO}$ and 0.15 mbar $P_{CO2}$. (a and b) The raw C 1s and Fe 2p NAP-XPS data of NAP-XPS test at 0.45 mbar $P_{CO}$ and 0.15 mbar $P_{CO2}$. (c) The deconvoluted C 1s XPS spectrum. (d) The deconvoluted Fe 2p XPS spectrum, including the satellite (sat.) peak. (e) The raw Co 2p XPS spectrum, including the satellite peak. All spectra were collected at different applied potentials, including OCV, at 0.45 mbar $P_{CO}$ and 0.15 mbar $P_{CO2}$.

Table S7. The NAP-XPS fitting results.

| Condition | Element | Applied Cell voltage | Percentage of respective species (%) |
|---|---|---|---|
| | Fe (0) | OCV | 6.5 |
| | | 1.5 V | 5.1 |
| | | 2.1 V | 5.3 |
| | | 2.7 V | 3.8 |
| | C-C | OCV | 0 |
| | | 1.5 V | 0 |
| | | 2.1 V | 0 |
| | | 2.7 V | 0 |
| | Fe (0) | OCV | 59.12 |
| | | 1.4 V | 51.08 |
| | | 1.9 V | 46.52 |
| | | 2.2 V | 39.33 |
| | C-C | OCV | 81.40 |
| | | 1.4 V | 56.10 |
| | | 1.9 V | 26.37 |
| | | 2.2 V | 24.57 |

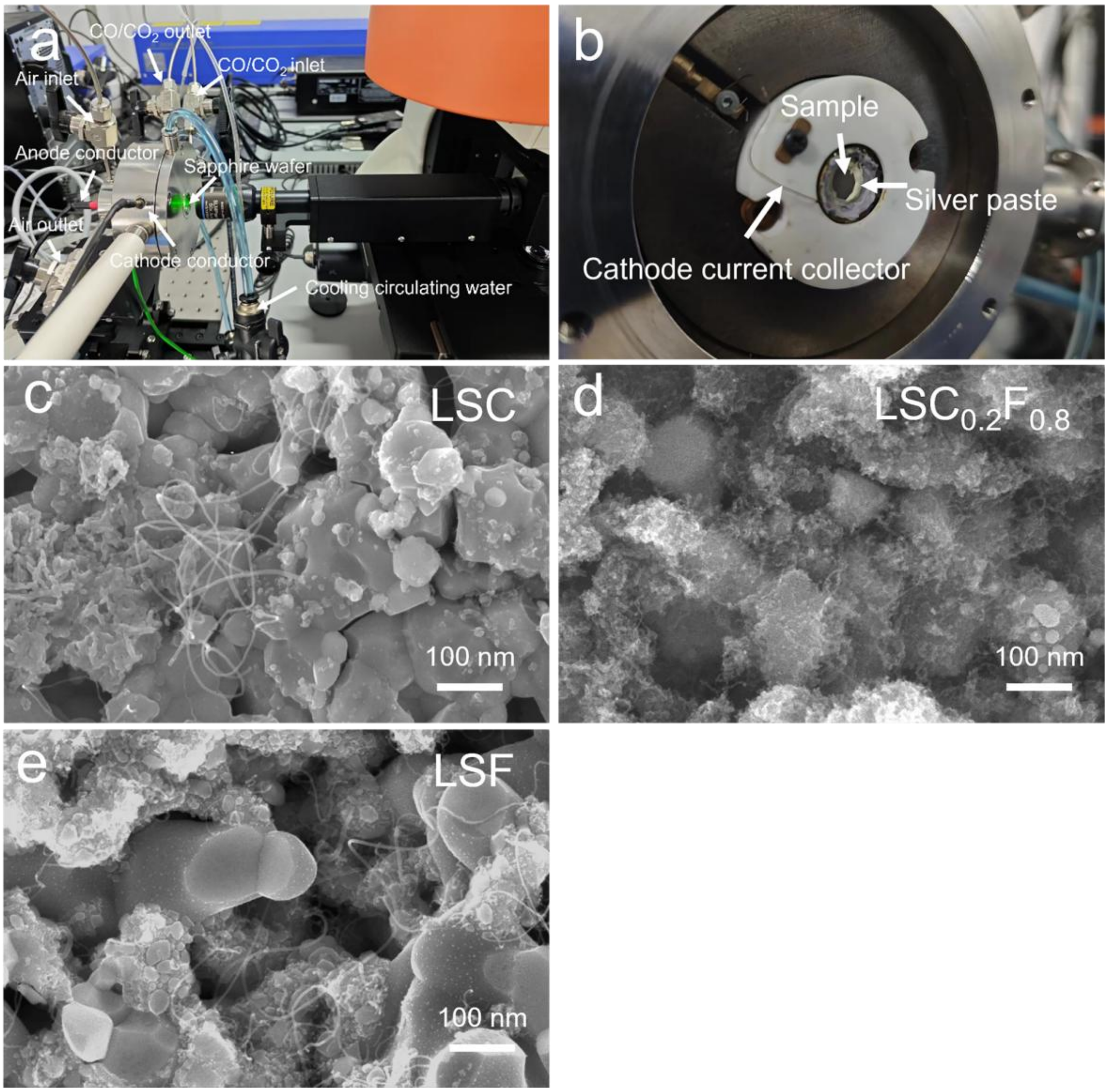


Figure S30. The quasi-in-situ Raman setup and SEM images of samples after the test. (a) The picture of the quasi-in-situ Raman setup. (b) The picture of the sample loaded in the quasi-in-situ Raman setup. (c-d) The SEM images of LSC, $LSC_{0.2}F_{0.8}$, and LSF without coke formation after quasi-in-situ Raman measurement.

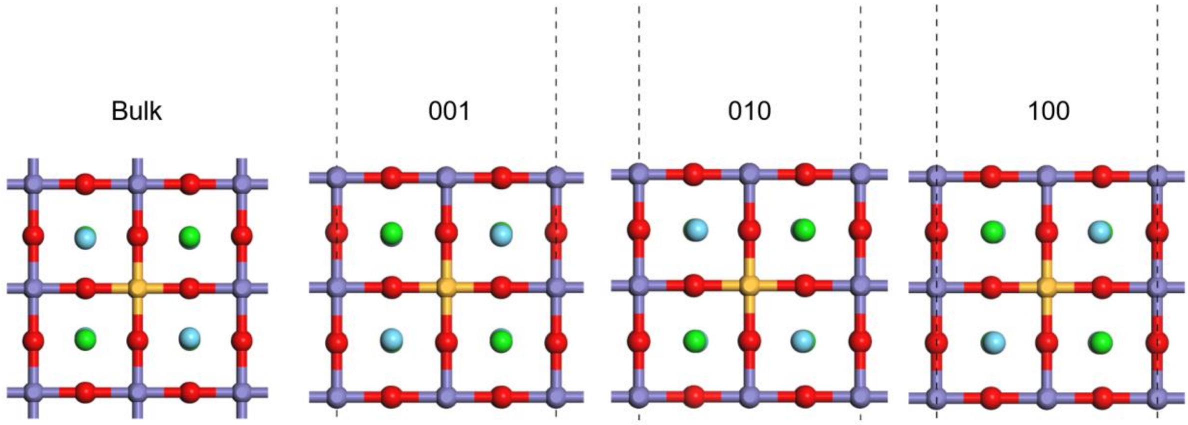

Figure S31. Structural illustration of Bulk LSCF and 001, 010, and 100 facets.

Table S8. All metal structures used in the DFT calculation.

| Metals | C* | CO* | $CO_2$* |
|---|---|---|---|
| Co(0001) | 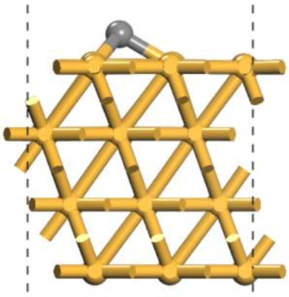 | 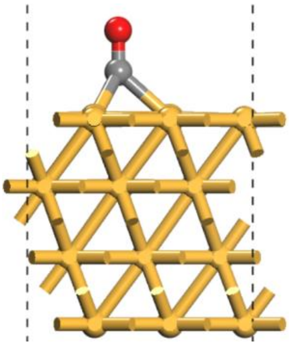 | 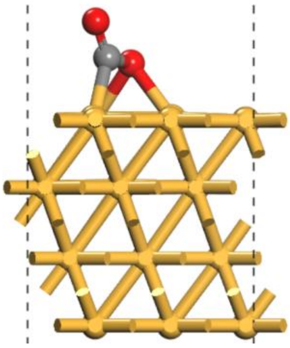 |
| Fe(100) | 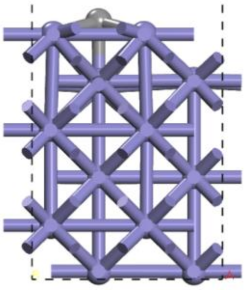 | 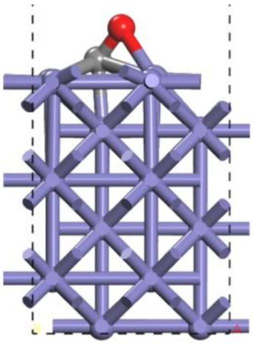 | 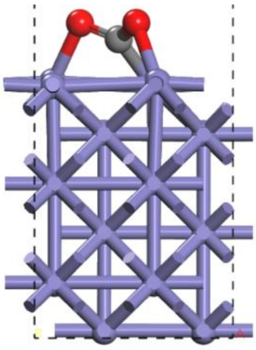 |
| Fe(110) | 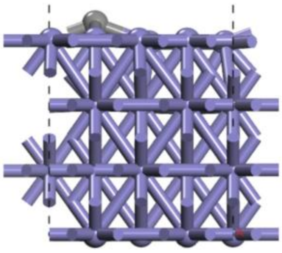 | 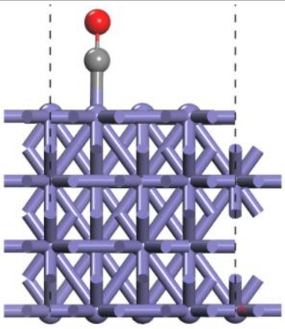 | 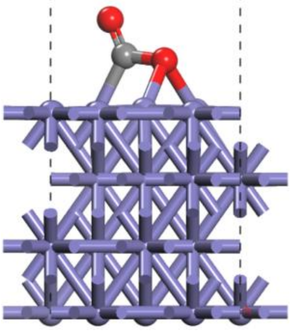 |
| Ni(111) | 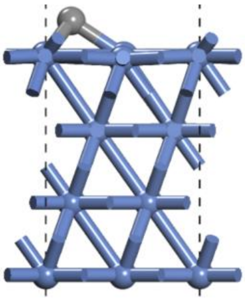 | 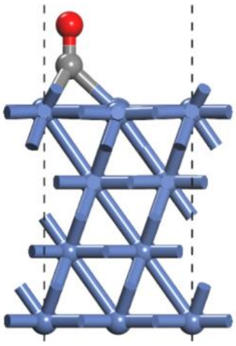 | 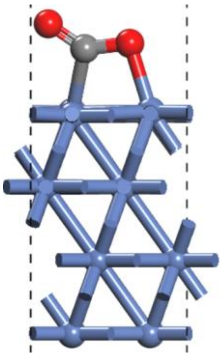 |

| | | | |
|---|---|---|---|
| Ni(211) | 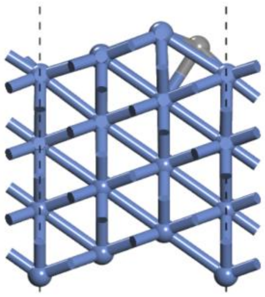 | 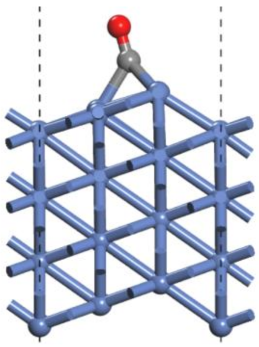 | 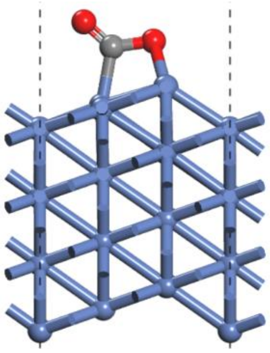 |
| FeCo(100) | 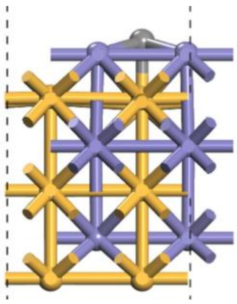 | 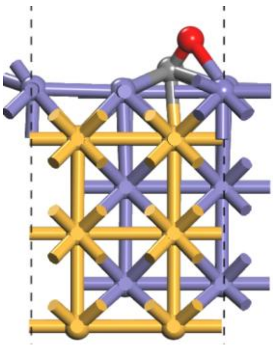 | 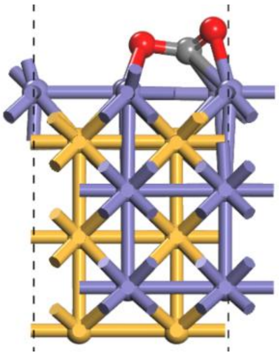 |
| FeCo(110) | 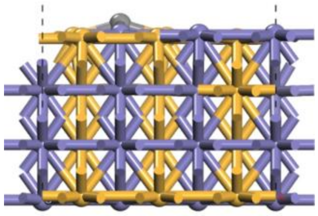 | 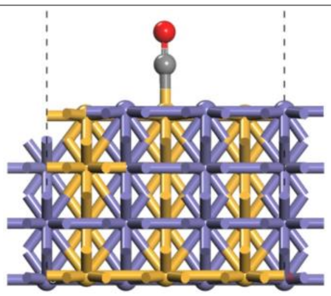 | 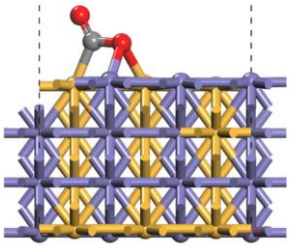 |

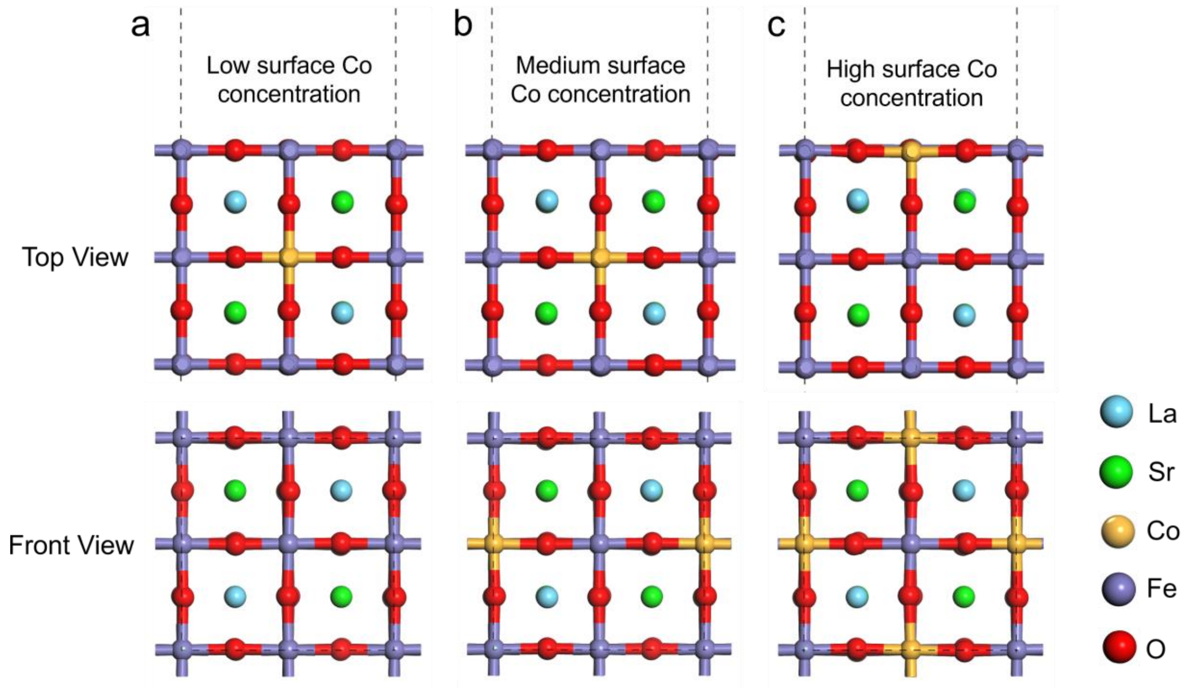


Figure S32. DFT models. Front and top views of (a) low, (b) medium, and (c) high surface Co concentration LSCF slab models.

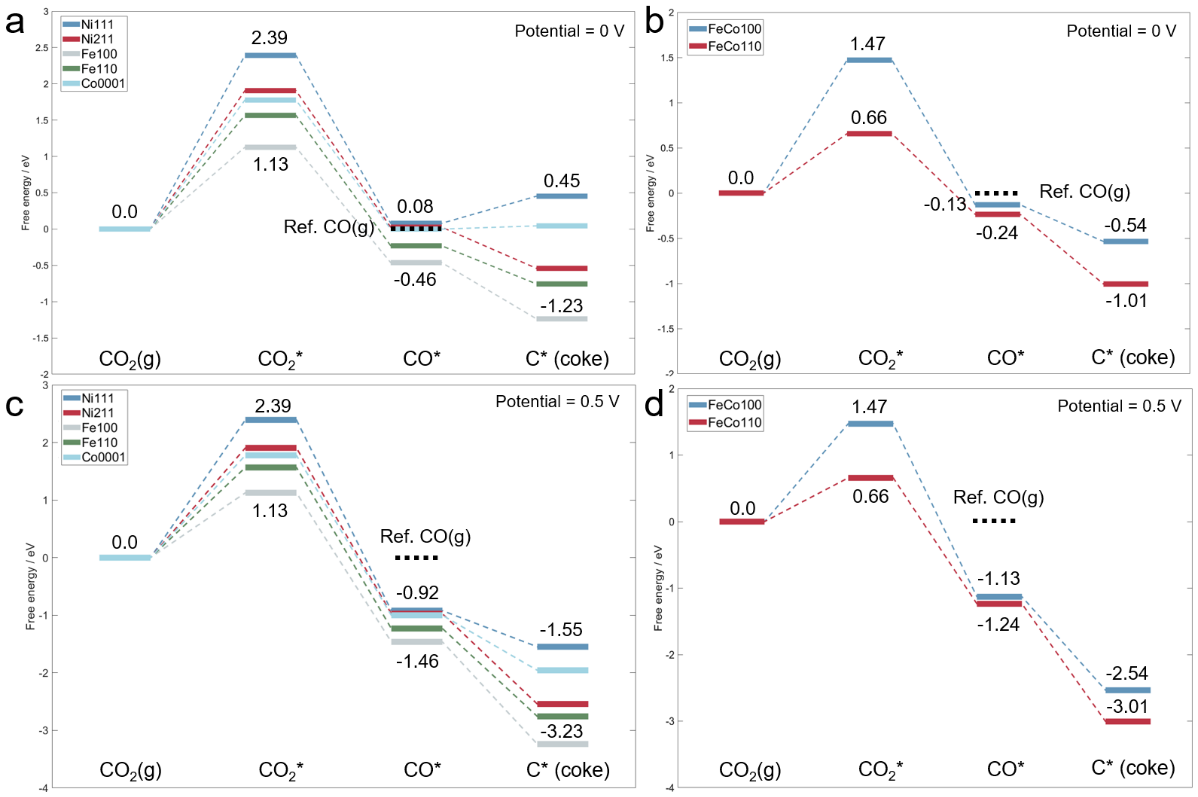


Figure S33. Free energy profile of $CO_2$ reduction to CO and coke formation on Ni, Fe, Co, and FeCo alloy metal surfaces. At a moderate potential of 0.5 V, the free energy of C* is already much lower than Ref. CO(g) and coke formation are inevitable.

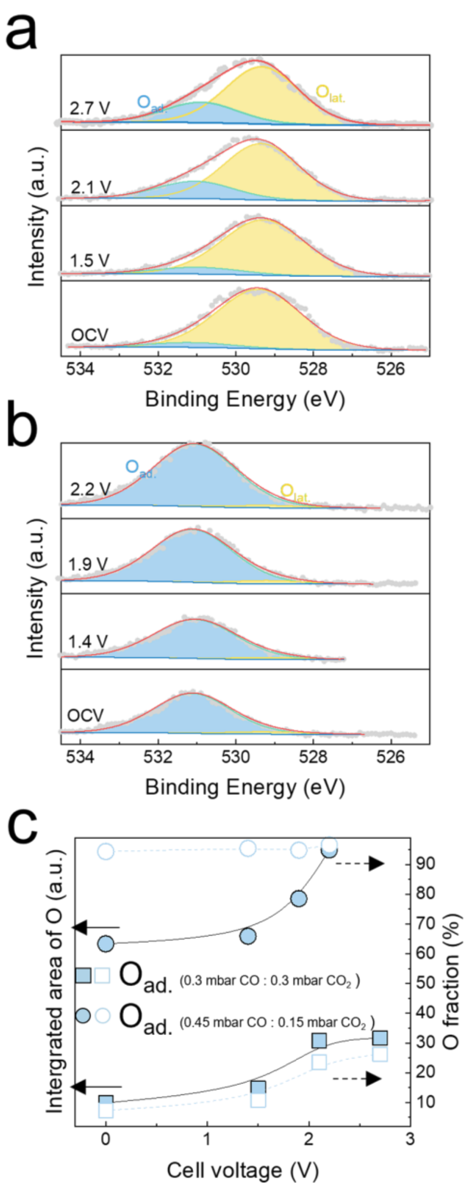


Figure S34. The O 1s NAP-XPS data. (a) The deconvoluted O 1s NAP-XPS spectra at 0.3 mbar $P_{CO}$ and 0.3 mbar $P_{CO2}$. (b) The deconvoluted O 1s NAP-XPS spectra at 0.45 mbar $P_{CO}$ and 0.15 mbar $P_{CO2}$. (c) The integrated area and the fraction of O-vacancies (absorbed O, $O_{ad}$) at different cell voltages.

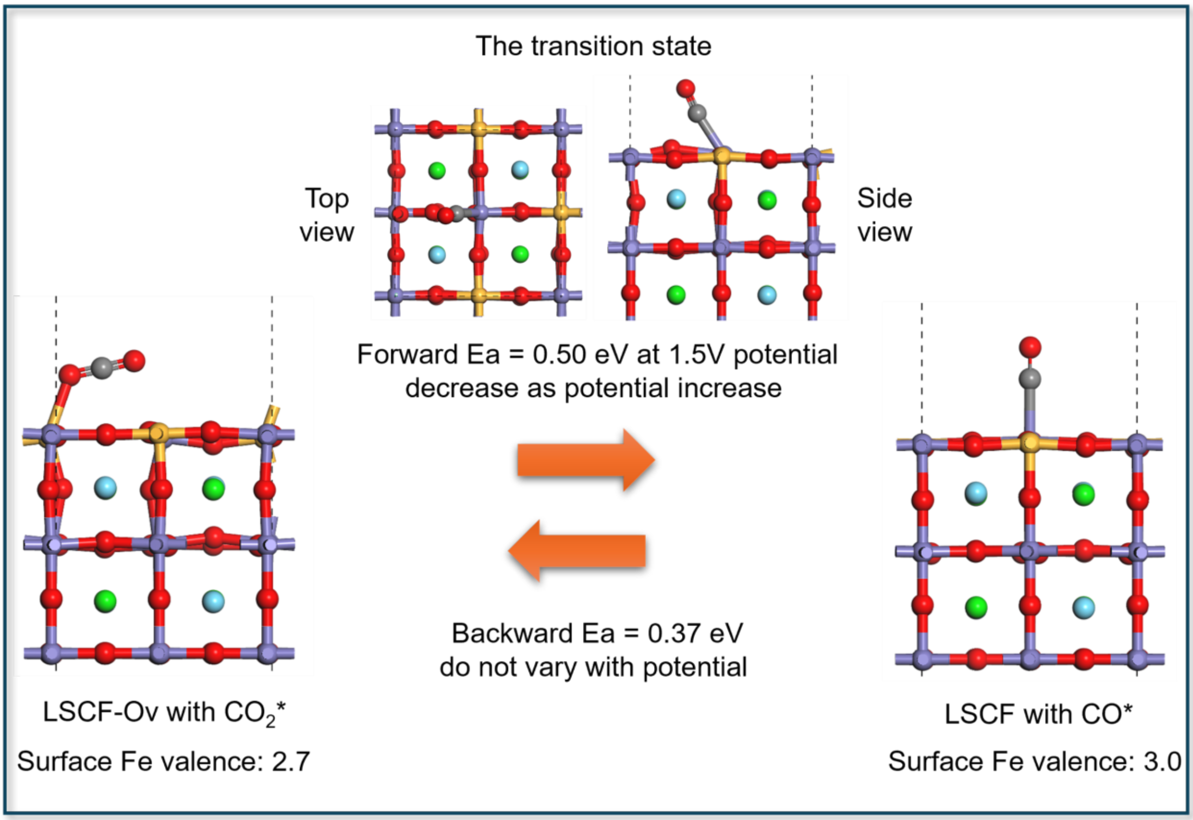


Figure S35. The transition state and forward/backward energy barriers of $CO_2$*/ LSCF -Ov → CO*/ LSCF.

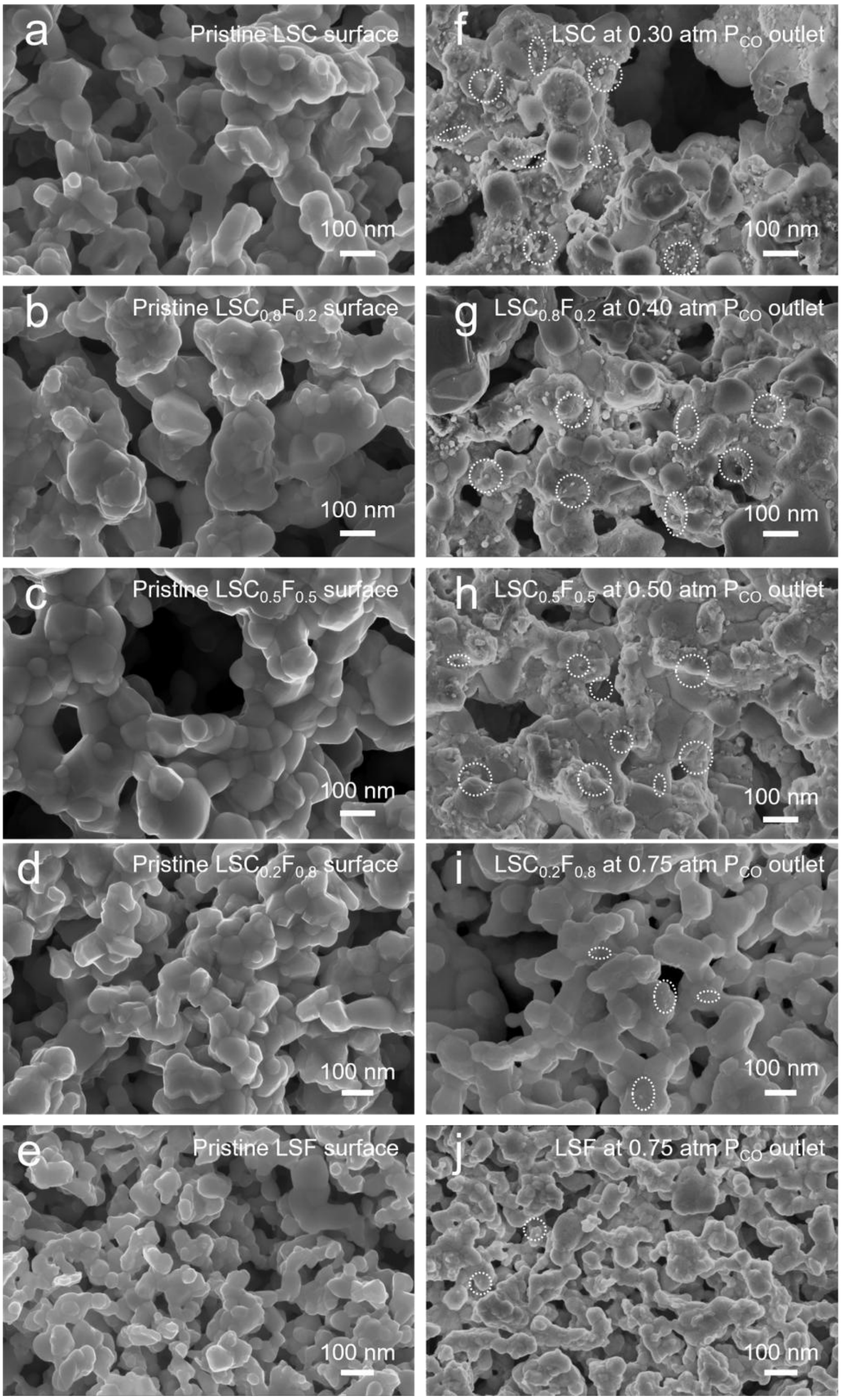


Figure S36. Morphology comparison of $LSC_{1-x}F_x$/GDC before and after the tests at different outlet $P_{CO}$. (a-e) The SEM images of the pristine $LSC_{1-x}F_x$/GDC electrodes. (f-j) The SEM images of $LSC_{1-x}F_x$/GDC after the test at 100 mA $cm^{-2}$ for 1 h. The outlet $P_{CO}$ is ~0.30 atm for LSC, ~0.40 atm for $LSC_{0.8}F_{0.2}$, ~0.50 atm for $LSC_{0.5}F_{0.5}$, and ~0.75 atm for $LSC_{0.2}F_{0.8}$ and LSF, respectively. The white circle highlights the exsolved metal or alloy nanoparticles. These images show that increasing the Fe fraction suppresses B-site metal exsolution.

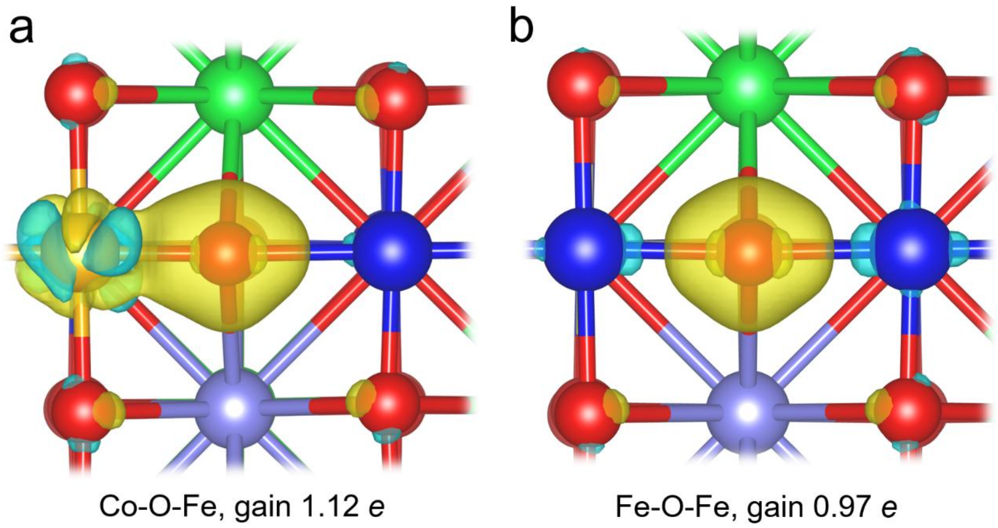


Figure S37. Charge density difference before and after Ov formation over LSCF with high surface Co concentration. (a) and low surface Co concentration (b). Yellow indicates electrons accumulation while blue represents depletion. The isosurface value is 0.01.

Table S9. Comparison of the performance of the sym-$LSC_{1-x}F_x$ /GDC cell and the $CO_2$-CO-$C_{2+}$ tandem to those reported previously.

| Cathode material | Stability/degradation rate/CO pressure | $CO_2$ SPU | CO concentration | Reference in the manuscript |
|---|---|---|---|---|
| $LSC_{0.2}F_{0.8}$/GDC | ~320 h/~0.09 mV $h^{-1}$/0.8 atm | 75±2% | 0.86±0.02 atm | This work |
| Ni/YSZ | ~1 h@~132 mV $h^{-1}$ (~3.00 V) and 0.77 atm | ~26% | 0.77 atm | *J. Power Sources* ***2015****, 276, 26-31* |
| LSCM/CMF | ~50 h @ ~1 mV $h^{-1}$ (~1.07 V) and 0.51 atm | ~11% | 0.73 atm | *Adv. Energy Mater.* ***2021****, 11, 2100339* |
| PSNFM-NFA@FeO | ~500 h@~0.84 mV $h^{-1}$ (~1.25 V) and 0.02 atm | ~5% | 0.05 atm | *Adv. Funct. Mater.* ***2022****, 32, 2202878* |
| CoFe@LSCFM/GDC | ~100 h @ ~0.54 mA $h^{-1}$ (1.2 V) and 0.05 atm | ~11% | 0.11 atm | *Angew. Chem. Int. Ed.* ***2020****, 59, 15968 – 15973* |
| SFRuM/GDC | ~1000 h @ ~0.16 mA $h^{-1}$ (1.2 V) and 0.05 atm | ~10% | 0.09 atm | *Nat. Commun.* ***2021****, 12, 5665* |
| SF1.5MC/GDC | ~80 h @ ~0.17 mA $h^{-1}$ (1.2 V) and 0.03 atm | ~10% | 0.1 atm | *Adv. Energy Mater.* ***2022****, 12, 2202175* |
| Fe-Ni/LSFN | ~100 h @ ~0.46 mA $h^{-1}$ (1.5 V) and 0.2 atm | ~36% | 0.55 atm | *ACS Catal.* ***2016****, 6, 6219-6228* |
| SFMC/GDC | ~230 h @ ~0.11 mA $h^{-1}$ (1.2 V) and 0.03 atm | ~10% | 0.09 atm | *Adv. Mater.* ***2020****, 32, 1906193* |

| The tandem stand | Stability@current and voltage | $CO_2$ SPU | Reference in the manuscript |
|---|---|---|---|
| Sym-$LSC_{0.2}F_{0.8}$ /GDC – CORR MEA | ~130 h@6 A and ~2.5 V<br>~60 h@10 A and ~2.8 V | 57±2% ($C_{2+}$ products)<br>27±1% ($C_2H_4$) | This work |
| Ni/YSZ – CORR MEA | ～40 h@0.6 A and 2.5 V | ～11% | *Joule* ***2021****, 5, 706-719* |

N.A. indicates the data is not available.

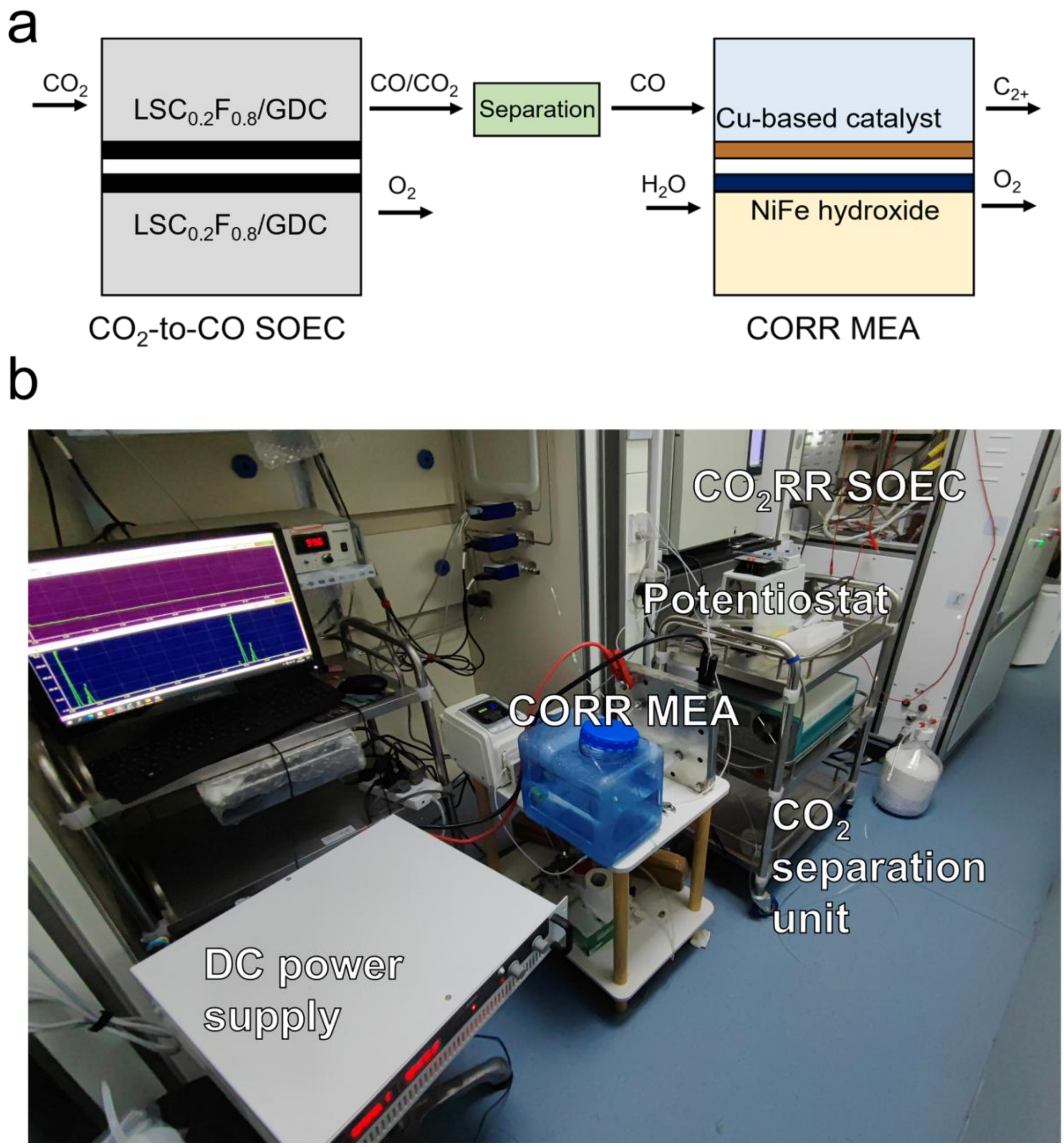


Figure S38. The $CO_2$-CO-$C_{2+}$ tandem stand. (a) The schematic illustration of the $CO_2$-CO-$C_{2+}$ tandem stand consists of an HT-$CO_2$RR SOEC and a CO electrolyzer. (b) The image of the tandem system.

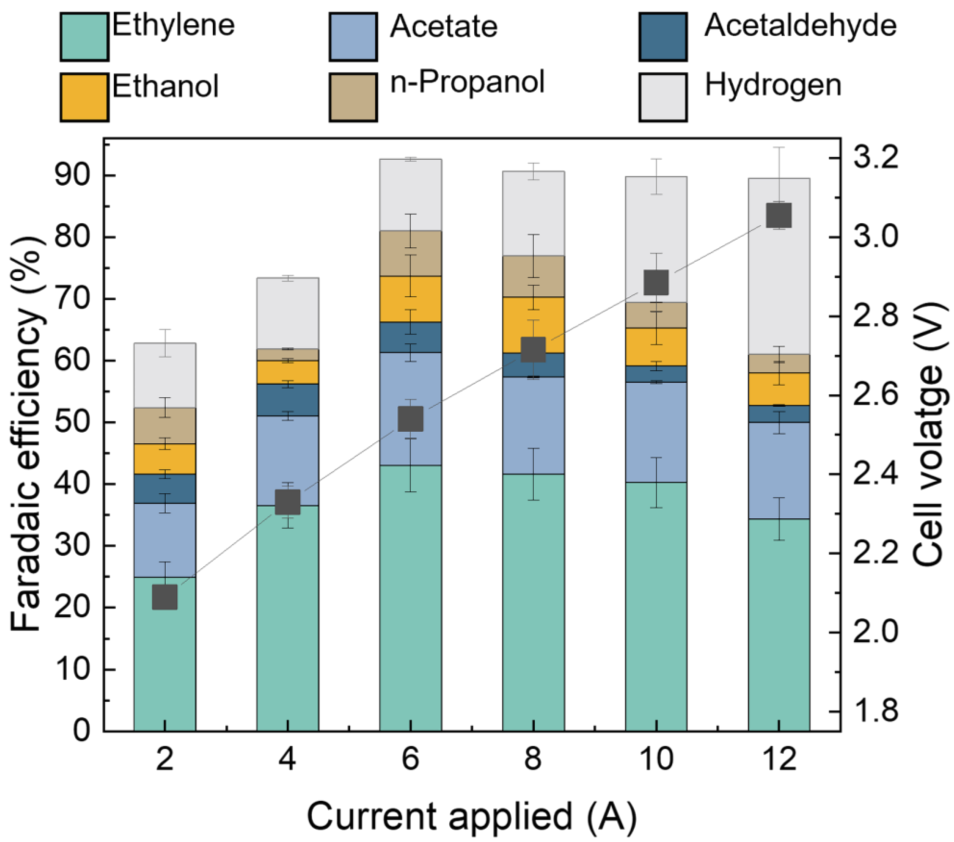


Figure S39. The CO reduction product FEs in the 100 $cm^2$ MEA reactor. Error bars correspond to standard deviations of three independent measurements.

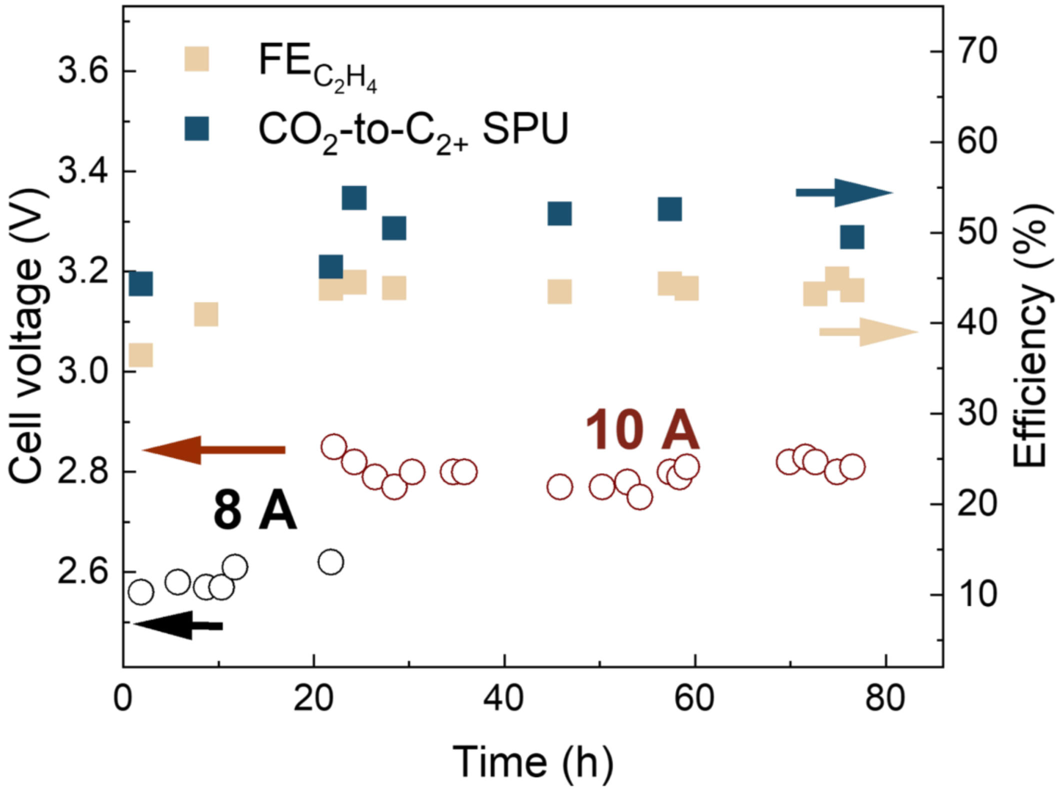


Figure S40. Stability of the cell voltage, $C_2H_4$ FE, and $CO_2$-to-$C_{2+}$ SPU at 8 and 10 A.

## References


[1] Overa, S.; Crandall, B. S.; Shrimant, B.; Tian, D.; Ko, B. H.; Shin, H.; Jiao, C. B. a. F. Enhancing acetate selectivity by coupling anodic oxidation to carbon monoxide electroreduction. *Nat. Catal.* **2022**, 5, 738-745.

[2] Duan, C.; Kee, R.; Zhu, H.; Sullivan, N.; Zhu, L.; Bian, L.; Jennings, D,; O'Hayre, R. Highly efficient reversible protonic ceramic electrochemical cells for power generation and fuel production. *Nat. Energy* **2019**, 4, 230-240.

[3] Chastain, J.; King Jr, R. C. Handbook of X-ray photoelectron spectroscopy. *Perkin-Elmer Corporation* **1992**, 40, 221.

[4] Perdew, J. P.; Burke K.; Ernzerhof, M. Generalised Gradient Approximation Made Simple. *Phys. Rev. Lett.* **1996**, 77, 3865-3868.

[5] Kresse, G.; Furthmüller, J. Efficiency of ab-initio total energy calculations for metals and semiconductors using a plane-wave basis set. *Comput. Mater. Sci.* **1996**, 6, 15-50.

[6] Kresse, G.; Hafner, J. Ab initio molecular-dynamics simulation of the liquid-metal-amorphous-semiconductor transition in germanium. *Phys. Rev. B* **1994**, 49, 14251-14269.

[7] Klimeš, J.; Bowler, D. R.; Michaelides, A. Van der Waals density functionals applied to solids. *Phys. Rev. B* **2011**, 83, 195131.

[8] Kresse, G.; Joubert, D. From ultrasoft pseudopotentials to the projector augmented-wave method. *Phys. Rev. B* **1999**, 59, 1758-1775.

[9] Blöchl, P. E. Improved tetrahedron method for Brillouin-zone integrations. *Phys. Rev. B* **1994**, 49, 16223-16233.

[10] Lv, H.; Lin, L.; Zhang, X.; Li, R.; Song, Y.; Matsumoto, H.; Ta, N.; Zeng, C.; Fu, Q.; Wang, G.; Bao, X. Promoting exsolution of RuFe alloy nanoparticles on $Sr_2Fe_{1.4}Ru_{0.1}Mo_{0.5}O_{6-\delta}$ via repeated redox manipulations for $CO_2$ electrolysis. *Nat. Commun.* **2021**, 12, 5665.

[11] Wei, M.; Li, H.; Guo, G.; Liu, Y.; Zhang, D. Effects of PdO modification on the performance of $La_{0.6}Sr_{0.4}Co_{0.2}Fe_{0.8}O_{3-\delta}$ cathodes for solid oxide fuel cells: A first principle study. *Int. J. Hydrogen Energy* **2017**, 42, 23180-23188.

[12] Jia, T.; Zeng, Z.; Zhang, X.; Ohodnicki, P.; Chorpening, B.; Hackett, G.; Lekse, J.; Duan, Y. The influence of oxygen vacancy on the electronic and optical properties of $ABO_{3-\delta}$ (A = La, Sr, B = Fe, Co) perovskites. *Phys. Chem. Chem. Phys.* **2019**, 21, 20454-20462.

[13] Tezel, E.; Guo, D.; Whitten, A.; Yarema, G.; Freire, M.; Denecke, R.; McEwen, J.-S.; Nikolla, E. Elucidating the Role of B-Site Cations toward $CO_2$ Reduction in Perovskite-Based Solid Oxide Electrolysis Cells. *J. Electrochem. Soc.* **2022**, 169, 034532.

[14] Xie, M.; Cai, C.; Liu, X.; Xue, K.; Chen, Y.; Peng, J.; Bao, J.; An, S.; Yang, H. Improved Durability of High-Performance Intermediate-Temperature Solid Oxide Fuel Cells with a Ba-Doped $La_{0.6}Sr_{0.4}Co_{0.2}Fe_{0.8}O_{3-\delta}$ Cathode. *ACS Appl. Mater. Interfaces* **2022**, 14, 33052-33063.

[15] Pei, K.; Zhou, Y.; Ding, Y.; Xu, K.; Zhang, H.; Yuan, W.; Sasaki, K.; Choi, Y.; Liu, M.; Chen, Y. An improved oxygen reduction reaction activity and $CO_2$-tolerance of $La_{0.6}Sr_{0.4}Co_{0.2}Fe_{0.8}O_{3-\delta}$ achieved by a surface modification with barium cobaltite coatings. *J. Power Sources* **2021**, 514, 230573.

[16] Chen, H.; Guo, Z.; Zhang, L. A.; Li, Y.; Li, F.; Zhang, Y.; Chen, Y.; Wang, X.; Yu, B.; Shi, J.-m. Improving the Electrocatalytic Activity and Durability of the $La_{0.6}Sr_{0.4}Co_{0.2}Fe_{0.8}O_{3-\delta}$ Cathode by Surface Modification. *ACS Appl. Mater. Interface* **2018**, 10, 39785-39793.

[17] Yu, Y.; Luo, H.; Cetin, D.; Lin, X.; Ludwig, K.; Pal, U.; Gopalan, S.; Basu, S. Effect of atmospheric $CO_2$ on surface segregation and phase formation in $La_{0.6}Sr_{0.4}Co_{0.2}Fe_{0.8}O_{3-\delta}$ thin films. *Appl. Surf. Sci.* **2014**, 323, 71-77.
[18] Stukowski, A. Visualization and analysis of atomistic simulation data with OVITO–the Open Visualization Tool. *Model. Simul. Mater. Sci.* **2010**, 18, 015012.
[19] Alavi, A.; Hu, P.; Deutsch, T.; Silvestrelli, P. L.; Hutter, J. CO Oxidation on Pt(111): An Ab Initio Density Functional Theory Study. *Phys. Rev. Lett.* **1998**, 80, 3650-3653.
[20] Liu, Z.-P.; Hu, P. General Rules for Predicting Where a Catalytic Reaction Should Occur on Metal Surfaces: A Density Functional Theory Study of C−H and C−O Bond Breaking/Making on Flat, Stepped, and Kinked Metal Surfaces. *J. Am. Chem. Soc.* **2003**, 125, 1958-1967.
[21] Wang, Z.; Wang, H.-F.; Hu, P. Possibility of designing catalysts beyond the traditional volcano curve: a theoretical framework for multi-phase surfaces. *Chem. Sci.* **2015**, 6, 5703-5711.
[22] Wang, Z.; Cao, X.-M.; Zhu, J.; Hu, P. Activity and coke formation of nickel and nickel carbide in dry reforming: A deactivation scheme from density functional theory. *J. Catal.* **2014**, 311, 469-480.
[23] Tang, W.; Sanville, E.; Henkelman, G. A grid-based Bader analysis algorithm without lattice bias. *J. Phys. Condens. Matter.* **2009**, 21, 084204.
